# Exploring the complex interplay between oral *Fusobacterium nucleatum* infection, periodontitis, and robust microRNA induction, including multiple known oncogenic miRNAs

**DOI:** 10.1128/msystems.01732-24

**Published:** 2025-06-25

**Authors:** Syam Jeepipalli, Aravindraja C, William Duncan, V. M. Krishna, Bikash Sahay, Edward K. L. Chan, L. Kesavalu

**Affiliations:** 1 Department of Periodontology, College of Dentistry, University of Florida 3463, Gainesville, Florida, USA; 2 Department of Community Dentistry and Behavioral Science, College of Dentistry, University of Florida 3463, Gainesville, Florida, USA; 3 Department of Infectious Diseases and Immunology, College of Veterinary Medicine, University of Florida 3463, Gainesville, Florida, USA; 4 Department of Oral Biology, College of Dentistry, University of Florida 3463, Gainesville, Florida, USA; Argonne National Laboratory, Lemont, Illinois, USA

**Keywords:** *F. nucleatum*, periodontal disease, miRNAs, CRC miRNAs, machine learning models

## Abstract

*Fusobacterium nucleatum* (*F. nucleatum*) is an oral commensal bacterium that can become pathogenic and is associated with periodontitis, adverse pregnancy outcomes, and colorectal cancer (CRC). MicroRNAs are conserved, non-coding RNA molecules that regulate gene expression and are detected in microbial infections. This study aims to characterize the microRNA expression kinetics in the mandibles of C57BL/6J mice infected with *F. nucleatum* for 8 and 16 weeks and to identify miRNAs as potential biomarkers using NanoString nCounter miRNA panels. Mice were divided into four groups: 8 weeks of infection, 16 weeks of infection, and their respective sham infection. *F. nucleatum*-infected mice showed 100% bacterial colonization on the gingival surface, along with a significant increase in alveolar bone resorption (*P* < 0.0001) and intravascular dissemination to the heart, indicating its invasive potential. Out of 577 miRNAs analyzed, seven miRNAs were upregulated, and two miRNAs were downregulated in the 8 weeks of infection group. In the 16 weeks of infection group, seven miRNAs were upregulated while 13 miRNAs were downregulated. Notably, miR-205, miR-210, and miR-199a-3p were differentially expressed at 8 weeks as well as miR-28 at 16 weeks and have been previously reported in human periodontitis. The 13 miRNAs induced by *F. nucleatum* (e.g., miR-361-5p) are linked to 13 multiple malignancies. In addition, miR-126-5p has been identified as a potential biomarker for patients with PD and cardiovascular disease. These results indicate that *F. nucleatum* induces several PD-related miRNAs and links them to systemic comorbidities. Furthermore, this study revealed *F. nucleatum* induction of 14 oncogenic miRNAs, and specifically, 12 miRNAs were linked with CRC.

**IMPORTANCE:**

Our study investigated oral commensal *Fusobacterium nucleatum*, a critical bacterium associated with gum disease, adverse pregnancy outcomes, and enriched several tumors, including colorectal cancer (CRC). Recently, microRNAs have emerged as critical players in the interactions between host and microbes, and many host functions have been reported to be regulated by miRNAs during infection. *F. nucleatum* oral infection in mice induced gum disease, disseminated to the heart, lungs, and several miRNAs. Elevated miR-361 expression was linked to multiple cancers. In addition, miR-126-5p expression has been reported as a potential biomarker in patients with periodontitis and coronary artery disease, indicating *F. nucleatum*’s virulence potential. The 13 miRNAs induced by *F. nucleatum* are linked to 13 multiple malignancies, including CRC. These results indicate that *F. nucleatum* acts as a potent cancer-causing bacterium. This study opens new avenues for exploring *F. nucleatum*’s role in gum disease and its link with cancer.

## INTRODUCTION

The ubiquitous oral symbiont *F. nucleatum* is a heterogeneous species with five known subspecies. It is the most predominant and abundant species in the oral cavity, present in both diseased and healthy individuals (1). As a dominant microbe in the periodontium, it is a periodontal bacterium, a non-spore-forming obligate anaerobe, that does not possess fimbriae, pili, or flagella, and is detected at higher levels in the oral cavity of periodontitis patients (2). It is known to coaggregate/bridge/synergize with various microbial species and is a prominent intermediate oral pathobiont in the physical interaction and microbial complexes between Gram-positive and Gram-negative late colonizing bacteria, such as *Porphyromonas gingivalis*, *Treponema denticola*, and *Tannerella forsythia* (3, 4). It is an intermediate colonizer that colonizes the tooth and epithelial surface (5, 6), often implicated in various extra-oral diseases (4). Previous *in vivo* subcutaneous co-infection (7) and oral co-infection studies (8) have demonstrated that the inclusion of *F. nucleatum* synergistically enhances bacterial virulence and disease severity. Prior chronic oral infection established *F. nucleatum* colonization in the oral cavity, induced significant humoral IgG and IgM antibody response, and resulted in significant ABR and detection of genomic DNA in systemic organs (heart, aorta, liver, kidney, lung), indicating bacteremia (9). In addition, vascular inflammation was detected by enhanced systemic cytokines (CD30L, IL-4, IL-12), oxidized LDL, and serum amyloid A, as well as an altered serum lipid profile (cholesterol, triglycerides, chylomicrons, VLDL, LDL, HDL) in infected mice. An altered aortic gene expression in infected ApoE^null^ hyperlipidemic mice further supports its virulence (9).

Several studies have also reported the association of *F. nucleatum* with pregnancy complications, including chorioamnionitis, spontaneous abortion, preterm birth, stillbirth, neonatal sepsis, and preeclampsia (10). Emerging reports in the last two decades indicate a significant link between *F. nucleatum* and the progression of multiple digestive tract cancers, including esophageal (11), gastric (12), pancreatic (13), CRC (14, 15), lung cancers (16), and oral squamous cell carcinoma (OSCC) (17). *F. nucleatum* interacts with the molecular hallmarks of gastrointestinal cancers, inducing genomic mutations and promoting a permissive immune microenvironment by impairing anti-tumor checkpoints (18). *F. nucleatum* binds to the stressed epithelial cells using the Gal-GalNAc moiety and spreads to the extraoral site by the hematogenous route (19–21).

microRNAs (miRNAs) are small (21–25 nucleotides) noncoding, regulatory RNAs that regulate gene expression by directly binding to the 3′ untranslated regions of their target miRNAs (22). Approximately 60% of all genes in each mammalian genome are estimated to be regulated by 2,000 miRNAs (23, 24). Several studies on miRNAs have implicated them in many human systemic diseases. They regulate the immune response of hosts infected with exogenous pathogenic bacteria such as *Helicobacter pylori* (25), *Treponema pallidum* (26), *M. tuberculosis* (27), *Salmonella* (28), *Listeria monocytogenes* (29), *Mycobacterium avium* (30), and endogenous periodontal microbiome (31–34). Hence, understanding miRNA expression patterns could lead to the development of novel diagnostic and therapeutic biomarkers for microbial infection-mediated inflammatory diseases, including periodontal disease (PD). Gingival tissue from chronic inflammatory PD has revealed a panel of microRNAs (hsa-miR-223-3p, hsa-miR-203b-5p, hsa-miR-146a-5p, hsa-miR-146b-5p, and hsa-miR-155-5p) (35) and miR-146a expression in a rodent model (36). Hence, understanding the expression pattern of miRNAs could potentially lead to the development of novel diagnostic biomarkers for PD. Recently, we reported sex-specific differential miRNA expression (miR-9, miR-148a, miR-669a, miR-199a-3p, miR-1274a, miR-377, and miR-690) in mice infected with partial human mouth microbes (PAHMM) using a novel ecological time-sequential polybacterial periodontal infection (ETSPPI) mouse model (34). In addition, we have also reported individual monobacterial periodontal infections with *P. gingivalis*, *T. denticola*, *T. forsythia*, and *S. gordonii* in the mouse model and identified several miRNAs linked with PD, various systemic diseases, and malignancies (31–33). To date, the role of miRNAs in response to oral infection by the periodontal microbe *F. nucleatum* in a mouse model of PD has not been examined. Accordingly, this study aimed to enhance our understanding of whether intraoral infection of mice with *F. nucleatum* could lead to unique alterations in miRNA expression patterns. The present study was designed to analyze miRNA differential expression (DE) kinetics at two time points (8 weeks and 16 weeks) in *F. nucleatum-*infected male and female C67BL/6J mice using high-throughput NanoString analysis with nCounter miRNA expression profiling. In addition to analyzing the complicated interactions between *F. nucleatum* infection, PD, and oncogenic miRNAs, we utilized machine-learning (ML) algorithms such as XG Boost (XGB), Random Forest classifier (RFC), Logistic Regression (LR), Support Vector Classifier (SVC), and Multilayer Perceptron (MLP). In recent studies, we analyzed miRNAs in response to oral monobacterial infection in the mouse model using several ML algorithms (33, 37), which clarifies the understanding of miRNAs’ association with PD, its systemic comorbidities, and multiple tumors.

## MATERIALS AND METHODS

### Animal models, ethical statement, and grouping

Male and female wild-type C57BL/J mice aged 8 weeks were purchased from the Jackson Laboratory. All the animal procedures were performed according to the guidelines of the University of Florida Institutional Animal Care and Use Committee protocol # 202200000223. Mice were divided into four groups (in each group, *n* = 10; five males and five females): Group I was infected with *F. nucleatum* for 8 weeks, Group II was sham infected for 8 weeks, Group III was infected with *F. nucleatum* for 16 weeks, and Group IV was sham-infected mice for 16 weeks (Table 1).

**TABLE 1:** Gingival plaque samples tested positive for *F. nucleatum* gDNA using PCR^*a*^

Group/bacteria (weeks)	Positive gingival plaque samples (*n* = 10)			
	8-week time point		16-week time point	
	2 weeks	4 weeks	6 weeks	12 weeks
Group I/*F. nucleatum* ATCC 49256 (8 weeks)	5/10	3/10	10/10	NC
Group II/sham infection (8 weeks)	0/10	NC	NC	NC
Group III/*F. nucleatum* ATCC 49256 (16 weeks)	6/10	9/10	10/10	NC
Group IV/sham infection (16 weeks)	0/10	NC	NC	0/10

^*a*^ Total numbers of gingival plaque samples collected after infections (2, 4, 6, and 12 weeks), and positive infections were determined by PCR analysis. NC, not collected to allow bacterial biofilm to adhere to the gingival surface, invade epithelial cells, and multiply. The first value corresponds to the number of mice that assessed positive for the respective genomic DNA, and the second value corresponds to the total number of mice in the group.

### Bacterial strain, culture, and mice-oral administration

*F. nucleatum* ATCC 49256 subspecies *vincentii* was isolated from periodontal pocket (38) grown on blood agar plates in a Coy anaerobic chamber at 37°C for 2–3 days, harvested, and prepared for intra-oral infections in mice as described previously (9, 34, 39–42). Mice in Group I were intraorally infected with 10^8^ *F. nucleatum* cells/mouse, suspended in reduced transport fluid (RTF) and equal volumes of carboxymethyl cellulose (6% CMC) for four times a week every other week for a total of 8 weeks. In Group III, the infection scheme was for a total of 16 weeks. Group II and IV mice were treated with a 1:1 ratio of RTF and 6% CMC as sham infections for 8 and 16 weeks, respectively. After the designated infection period, mice were euthanized (CO_2_ inhalation), and blood and organ samples (mandibles, maxilla, brain, heart, liver, lungs, spleen, and kidney) were collected. RNAlater solution was used to preserve the left maxilla and mandibles for miRNA analysis, whereas the right maxilla and mandibles were used for horizontal alveolar bone resorption (ABR) morphometry measurements (34).

### DNA isolation and molecular detection of the bacteria genome

Gingival plaque samples from the mice that received oral *F. nucleatum* infection were collected in Tris EDTA (TE) buffer (34, 43). *F. nucleatum* genomic DNA (gDNA) present in the oral plaque samples and the distal organs of the heart, lungs, brain, liver, kidney, and spleen were detected using 16S rRNA gene-specific primers 5′-TAAAGCGCGTCTAGGTGGTT-3′ and reverse primer 5′-ACAGCTTTGCGACTCTCTGT-3′ (34). Colony PCR was adopted to detect *F. nucleatum* gDNA in the gingival plaque samples. Qiagen Dneasy Blood and Tissue Kit (Qiagen, Germantown, MD, USA) was used to extract the gDNA from the distal organs and stored at –20°C (34). *F. nucleatum* culture DNA was used as a positive control, and sterile milli Q water was considered a negative control in the PCR. The amplified *F. nucleatum*-specific DNA was run through agarose electrophoresis and visualized in the UVP GelStudio touch Imaging System (Analytik Jena US LLC, CA, USA).

### Measurement of ABR

After euthanasia, the mandibles and maxilla were dissected and placed in a beaker, autoclaved to remove the soft flesh over the jawbone. Two-dimensional alveolar bone imaging was performed using a stereo dissecting microscope (Stereo Discovery V8, Carl Zeiss Microimaging, Inc., Thornwood, NY, USA). The pattern of the horizontal ABR area was measured by histomorphometry, as described previously (34, 43). Two examiners were blinded to measure ABR, and the data acquired were used for quantitative analysis.

### Total RNA isolation and quality assessment

Total RNA was isolated from left mandibles using the mirVana miRNA Isolation Kit (Ambion, Austin, TX, USA). The final RNA yield, quality assessments, and purity were determined as follows (31, 32, 34): RNA with an OD 260/230 ratio of >2 and an OD 260/280 ratio of >2 was quantified using an Epoch Microplate Spectrophotometer (BioTek, USA, Winooski, VT, USA) and taken for NanoString analysis.

### miRNA-transcriptome expression profiling

We used high-throughput nCounter expression panels (NanoString Technologies, Seattle, WA, USA) for miRNA-transcriptome analysis in the mandibles of the mice. Panels can identify 577 miRNAs in the sample using molecular barcodes and can detect even a low number of miRNAs without the need for reverse transcription (RT) or amplification. The probability of introducing artifacts is limited as it does not involve cDNA conversion as in Real-Time PCR. The NanoString nCounter Mouse miRNA Assay kit v1.5 is a highly sensitive multiplexed method that detects miRNA using nCounter reporter probes. This step does not require reverse transcription. The details of the NanoString experimental procedure were explained in detail from our previous publications (31–34, 37). Briefly, NanoString’s nCounter utilizes a chemistry that distinguishes miRNAs with single-nucleotide differences and has a 6-log dynamic range for direct digital counts. Accordingly, the process of reverse transcription is not required. nCounter miRNA expression assay is highly reproducible with R² >0.99 in replicate counts. To verify the sensitivity and specificity, we have performed the q-PCR validation of the inflammatory miRNA 146a and found that both the q-PCR and NanoString data were similar (41). Two independent studies also support the idea that there is no need to validate the NanoString data using stem-loop Real-Time PCR. The study from Prokopec et al. compared two key medium-throughput platforms—NanoString’s nCounter Analysis System and ABI’s OpenArray System—to gold-standard quantitative real-time RT-PCR for the specific factors signal:noise ratios, correlations, dynamic range, and detection accuracy comparison across the three platforms and found that all three measurement technologies showed good concordance but with divergent price/time/sensitivity trade-offs (44). In another independent study, Veldman-Jones et al. reported that NanoString nCounter shows excellent technical reproducibility (45).

The nCounter Mouse miRNA Assay kit v1.5 provided six positive hybridization controls and eight negative control probes to monitor hybridization efficiency. Twelve samples per cartridge were processed in a single run, which took 3 h. This was followed by digital analysis, which involved the transfer of the cartridge to the multichannel epifluorescence digital analyzer. A cartridge definition file with a maximum field of view (FOV) count of 555 per flow cell was taken for digital analysis. The number of images taken per scan corresponded to the number of immobilized reporter probes on the cartridge. A separate Reporter Code Count (RCC) file for each sample containing the count for each probe was downloaded and used for data analysis. The final Reporter Code Count (RCC) data files were evaluated using nSolver 4.0 software as described (31–34, 37). The data that support the findings of this study are openly available at https://www.ncbi.nlm.nih.gov/geo/query/acc.cgi?acc=GSM7915885 (accessed on 4 December 2023).

### Bioinformatics analysis

The normalized miRNA data were analyzed using the HyperScale architecture developed by ROSALIND, Inc. (San Diego, CA) (https://rosalind.bio/ (accessed on 16 September 2023) (46). Fold changes in the miRNA were calculated following the standard formula (33) and limma R library (47). The DE miRNAs were validated for miRNA-target gene interactions using the MiRTarBase database (48). A Venn diagram for the DE miRNAs was drawn using Venny 2.1 (34).

### Kyoto Encyclopedia of Genes and Genomes

Kyoto Encyclopedia of Genes and Genomes (KEGG) is an integrated database (49), and its pathways were plotted using the DIANA-miRPath v.3.0 database (50), taking the MIMAT accession number, calculating the false discovery rate (FDR) using the Benjamini and Hochberg method (34).

### Multiple ML model analysis

The ML models used in our study were created and executed using version 3.11.4 of the Python programming language. We used XGBoost version 1.7.6 and the Scikit-learn version 1.3.0 implementations of LR, SVC, MLP, and RFC. A full list of the hyperparameters used for each ML model is available in Appendix S1 in the Supplemental material. SHAP version 0.42.1 was used to obtain feature importance results (37). The code for executing the ML models on the NanoString copy data is available on GitHub at (https://github.com/uflcod/miRNA-periodontal-disease (accessed on 1 February 2024) in the “notebooks” directory. The notebooks for analyzing the *F. nucleatum* NanoString data begin with the prefix “*Fn*_” (e.g., *Fn*_randomforest_miRNA.ipynb, *Fn*_xgboost_miRNA.ipynb).

### Statistical analysis

All the data in the graphs were presented as mean ± SEM. Data for alveolar bone resorption were analyzed using ordinary two-way ANOVA, with Tukey’s multiple comparison test with a single pooled variance in Prism 9.4.1 (GraphPad Software, San Diego, CA, USA) (34, 37, 51). *P*-value < 0.05 was considered statistically significant. The DE miRNAs with FC of ±1 were considered significant. The identification of significant DE-miRNA was done based on two-tailed *t*-tests. The Welch–Satterthwaite equation was used to calculate the distribution of the *t*-statistics. The volcano plot was drawn using GraphPad Software (34).

## RESULTS

### Chronic infection of *F. nucleatum*, colonized in the mice gingival tissue

Intraoral infection of *F. nucleatum* induced a time-dependent colonization on the gingiva and aided periodontal miRNA expression in periodontitis. Time-dependent gingival colonization for *P. gingivalis*, *T. denticola*, *T. forsythia,* and *S. gordonii* was observed in our earlier studies (31–33, 37). *F. nucleatum* bacterial colonization initiated PD on chronic intraoral infection in a time-dependent manner. Testing of oral swabs collected from the gingival plaque for 16S rRNA gene-specific PCR confirmed that gingival plaque samples in Group I mice had 50% *F*. *nucleatum*-specific DNA at the 2 weeks of intraoral infection time point and tested positive for all the mice after 6 weeks of *F. nucleatum* infection. Group III mice were 60%, 90%, and 100% positive for *F. nucleatum* after 2, 4, and 6 weeks of infections, respectively (Table 1).

### Alveolar bone resorption and bacterial genomic DNA (gDNA) in the distal organs

**Fig 1:**
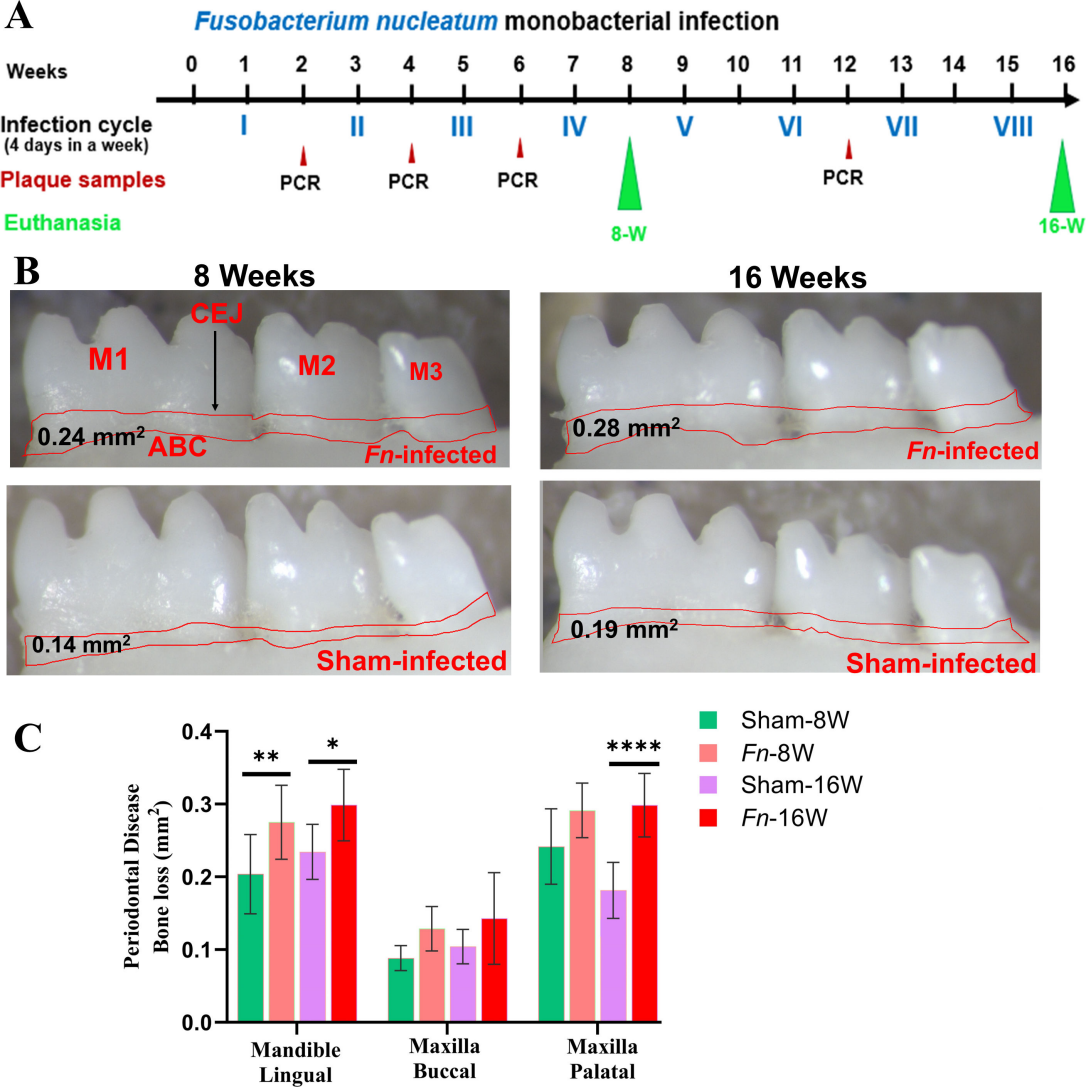
Intraoral infection with *F. nucleatum* significantly induced ABR. (**A**) Schematic diagram of the experimental design depicting the monobacterial infection with *F. nucleatum* (4 days per week on every alternate week), plaque sampling for PCR, and euthanasia. (**B**) Representative images showing horizontal ABR in the mandible (lingual view) of *F. nucleatum-*infected and sham-infected mice, with the ABR area outlined from the alveolar bone crest (ABC) to the cementoenamel junction (CEJ). (**C**) Morphometric analysis of the mandible and maxillary ABR at 8 and 16 weeks post-infection. A significant increase in ABR was seen in *F. nucleatum*-infected mice compared to sham-infected mice (****P < 0.0001; **P < 0.01; *P < 0.05; ordinary two-way ANOVA). Data points and error bars are represented as mean ± SEM (*n* = 10).

The pathogenic factors of periodontal bacteria, along with inflammatory cytokines mediated activation of osteoclasts, collectively contribute to ABR (52). Microscopic images of the mandibles in *F. nucleatum*-infected and sham-infected groups are shown in Fig. 1B. Mice infected with *F. nucleatum* at both 8 week and 16 week time points showed significantly higher ABR in the mandible (lingual) *P* < 0.05 (adjusted *P*-value = 0.001) (Fig. 1B and C). ABR measured in the maxilla did not show a significant difference between sham- versus *F. nucleatum*-infected groups. In the 8 weeks infection group, bacteria-specific gDNA was identified from the heart (Table S1) (3/10), lungs (2/10), and the liver (1/10). Similarly, in 16 weeks of infected mice, *F. nucleatum* gDNA was detected in the heart (5/10), the lungs (6/10), and the kidney (1/10) samples (Table S1). This finding suggests the physiological colonization/infection of the gingival epithelium and the intravascular dissemination of bacteria to the distal organs, representing the invasive potential of *F. nucleatum*.

### NanoString analysis of miRNA in *F. nucleatum*-infected mandibles

The NanoString platform is an amplification-free technology that uses molecular barcodes. It directly quantifies the RNA molecules without the events of reverse transcription and is strongly reliable in working with multiple sample types. The total RNA extracted from purified mandibles was further analyzed for global miRNA profiling in the 8 weeks and 16 weeks of *F. nucleatum*-infected mice (Table 2). nCounter miRNA expression profiling showed seven upregulated miRNAs, including most significantly miR-361-5p, miR-26a-5p, miR193a-3p, miR-126-5p, miR-324-5p, miR-24-3p, and miR-99b-5p and two downregulated miRNAs of miR-362-3p and miR-720 in 8 weeks of *F. nucleatum*-infected mandibles compared to sham-infected mandibles (Table 2). Similarly, a total of seven upregulated DE miRNAs (miR-361-5p, miR-99b-5p) and thirteen downregulated miRNAs (miR-323-3p, miR-488) were shown in 16 weeks of *F. nucleatum-*infected mandibles compared to the sham-infected mandibles. The analysis between *F. nucleatum*-infected female vs male mice showed 12 upregulated (miR-206, miR-210) and 12 downregulated miRNAs (miR-376a, miR-350) in the 8 weeks of infection group and 5 upregulated (miR-152, miR-125b-5p) and 14 downregulated miRNAs (miR-375, miR-376a) in the 16 weeks of infection group. The upregulated miR-210 in the 8 weeks of *F. nucleatum*-infected female mice was found to be downregulated in the 16 weeks of infected female mice (Table 2). A *P*-value of <0.05 and a fold change of 1.1 and above were considered for analysis and to be significant. DE upregulated miRNAs between 8 and 16 weeks of analysis target function and target genes are shown in Tables 3 and 4, and downregulated miRNAs are shown in Table S2. The list of DE miRNAs (upregulated and downregulated) for 8 and 16 weeks of analysis is shown in Table S3. The upregulated and downregulated miRNAs in the female vs male comparison study for the 8 weeks of infection are shown in Table S4 and for 16 weeks shown in Table S5.

**TABLE 2:** Differentially expressed miRs during 8 and 16 weeks of *F. nucleatum* infection in mice^*a*^

Weeks, infection, and sex	Upregulated miRNAs (*P* < 0.05)	Downregulated miRNAs (*P* < 0.05)
8 weeks—*F. nucleatum-*infected vs 8 weeks—sham infection (*n* = 10)	7 (e.g., miR-361-5p, miR-99b-5p)	2 (miR-362-3p, miR-720)
8 weeks—*F. nucleatum-*infected female vs male (*n* = 5)	12 (e.g., miR-206, miR-210)	12 (e.g., miR-376a, miR-350)
16 weeks—*F. nucleatum-*infected vs 16 weeks—sham infection (*n* = 10)	7 (e.g., miR-361-5p, miR-99b-5p)	13 (e.g., miR-323-3p, miR-488, miR-350)
16 weeks*—F. nucleatum-*infected female vs male (*n* = 5)	5 (e.g., miR-152, miR-125b-5p)	14 (e.g., miR-375, miR-210, miR-376a, miR-362-3p)
8 weeks—*F. nucleatum-*infected vs 16 weeks—*F. nucleatum-*infected (*n* = 10)	14 (e.g., miR-133a, miR-22)	47 (e.g., miR-323-3p, miR-1902)

^*a*^ The number of DE miRNAs was shown for *F. nucleatum-*infected mice after 8 and 16 weeks of infections. The commonly expressed miRNAs between 8 and 16 weeks of bacterial-infected groups are shown in brackets. Two miRNAs expressed in bacterial-infected groups were unique and specific to the 8 and 16 weeks of infections.

**TABLE 3:** *F. nucleatum*-infection-induced upregulated miRNAs (8 weeks), reported functions, and target genes^*a*^

miR (FC)	*P-*value	Reported functions	Target genes
**miR-361-5p** (1.3)	0.0311	Downregulated in the **13 types of cancer**, including **CRC** (53). Upregulated in stable coronary heart disease, acute coronary heart syndrome (54).	16 (e.g., *Ctbp2, Tfam, Nol7*)
**miR-26a-5p** (1.23)	0.0008	Downregulated in the *gingiva of periodontitis patients* (55). Associated with cardiovascular diseases (56). Associated with **CRC** (57).	426 (e.g., *Kpna2, Nus1, Rgs17*)
**miR193a -3p** (1.21)	0.0150	Downregulated in plasma and salivary exosomes of *Chronic periodontitis patients* (58). Downregulated in the *P. gingivalis-*LPS-treated human periodontal ligament cells (59). Dysregulated in **pancreatic cancer** (60), **colorectal cancer** (61), and **endometrial cancer** (62). A novel biomarker for Alzheimer’s disease diagnosis (63). Downregulated in the microbiota-mediated ulcerative colitis (64).	8 (e.g., *Gla, Ifngr2, Metap2*)
**miR-126-5p** (1.2)	0.0308	Upregulated in *T. denticola-induced periodontitis* (32). Preventing alveolar bone resorption in diabetic *periodontitis* (65). Upregulated miR in the *gingiva of PD patients* (66). Associated with **colorectal cancer** (67), promoting chemoresistance of **ovarian cancer** cells (68). Upregulated in coronary artery ectasia patients (69). Upregulated in thoracic aorta aneurysm patients (70). Reported as a molecular target for myocardial infarction treatment (71). Reported as a poultry meat quality and food safety marker miRNA (72).	55 (e.g., *Gfpt1, H2-D1, Il10rb, Kras*)
**miR-324-5p** (1.18)	0.0164	Downregulated in the *gingival tissue of periodontitis patients* (73). Reported as a therapeutic miR in **cervical cancer**, **colorectal cancer, gastric cancer, brain tumors, and hepatocellular carcinoma** (74). Diagnostic miRNA in the human cases with heart failure (75). Downregulated in osteoporosis patients (76).	12 (E.g., *Zfp295, Zscan12, Kcnk6*)
**miR-24-3p** (1.17)	0.0160	Downregulated in the unstimulated saliva of *chronic periodontitis patients* (58). Reported as a defensive miR against *periodontal inflammation* (77). A diagnostic marker for multiple cancers (78).	375 (e.g., *Oxt, Chrna1, Birc5*)
**miR-99b-5p** (1.14)	0.0120	Downregulated in the *gingiva of periodontitis patients* (73). Upregulated in the *P. gingivalis-induced periodontitis* (31). Downregulated in the saliva of chronic *periodontitis patients* (58). Upregulated in *M. tuberculosis*-infected murine dendritic cells (79). Novel chemosensitizing miRNAs in high-risk neuroblastoma (80, 81). Associated with the **cancer conditions to ovaries** (82), **prostate** (83), **colorectal** (84), **gastric** (85), **liver** (86), and **lungs** (87).	4 (e.g., *Comp, Grik3, Slc35d2*)

^*a*^ The upregulated miRNAs for 8 weeks of *F. nucleatum* infection were associated with aggressive periodontitis in human subjects’ saliva and gingival tissue (e.g., miR-26a-5p; miR-193a-3p). Bold indicates miRNAs associated with multiple cancers, and italics indicate miRNAs associated with periodontitis details of the biological.

**TABLE 4:** *F. nucleatum* infection induced upregulated miRNAs (16 weeks), reported functions, and target genes^*a*^

miR (FC)	*P-*value	Reported functions	Target genes
**let-7a-5p** (1.28)	0.0011	Downregulated in *T. denticola-induced periodontitis* (32). Downregulated in the saliva of patients with *aggressive periodontitis* (88). Upregulated in *gingival tissue* of *chronic periodontitis patients* (89). IL-13, a cytokine essential for allergic lung diseases, is regulated by mmu-let-7a-5p (90). Downregulated in bronchial biopsy of severe asthma patients (91). Associated with **CRC** (92).	28 (e.g., *Lin28a, IL6, Hoxa9*)
**miR-127-3p** (1.28)	0.0217	Upregulated in *T. forsythia-induced rodent periodontitis models* (33). Upregulated in the inflamed primary human gingival fibroblasts (93). Upregulated in the human advanced carotid atheroma (94). May play an important role in acute myocardial injury (95). Tumor suppressor miRNA in **triple-negative breast cancer cells** (96). Associated with **CRC** (97).	10 (e.g., *Rtl1, Gpi1, Ghdc*)
miR-3615p (1.19)	0.0357	Reported in 8 weeks of analysis study	
**miR-345-5p** (1.16)	0.0034	Reliable biomarker in patients with **oral squamous cell carcinoma** (98). Acting as an anti-inflammatory miRNA in mice with allergic rhinitis (99). Reported as a protective miRNA during gestational diabetes mellitus subjects (100). Associated with **CRC** (101).	10 (e.g., *Ccdc127, Eaf1, Atic*)
**let-7f-5p** (1.16)	0.0298	Upregulated in *human periodontitis gingival tissue* (66). Potential biomarker for abdominal aortic aneurysm (102). Involvement in the pathogenesis of SLE-lupus nephritis (103). Associated in **CRC** (92).	16 (e.g., *Atp2b2, Ifnar1, Nf2*).
miR-99b-5p (1.15)	0.0299	Reported in 8 weeks of analysis study	
**miR-218-5p** (1.15)	0.0333	Upregulated in the *T. forsythia-induced rodent periodontitis models* (33). Downregulated in the *gingival fibroblasts* and is essential for myofibroblast differentiation (104). Upregulated in the *inflamed gingiva* (105). Reduced expression was observed in the atherosclerosis cohort and considered a clinical marker for atherosclerosis (106). Downregulated in smokers without airflow limitation and in patients with chronic obstructive pulmonary disease (107). Associated with **CRC** (108).	20 (e.g., *Epg5, Prdm1, Bsn, Eno2*)

^*a*^ Details of the biological function and target genes were given for the top 10 significantly expressed miRNAs in 16 weeks of infected mice mandibles. The upregulated miRNAs for 16 weeks of *F. nucleatum* infection were associated with aggressive periodontitis in the saliva and gingival tissue of human subjects (mmu-let-7a-5p; mmu-let-7f-5p), acute myocardial injury (miR-127-3p), and a clinical marker for atherosclerosis (miR-218-5p). Bold indicates miRNAs associated with multiple cancers, and italics indicate miRNAs associated with periodontitis.

### Identification of differentially expressed miRNAs

**Fig 2:**
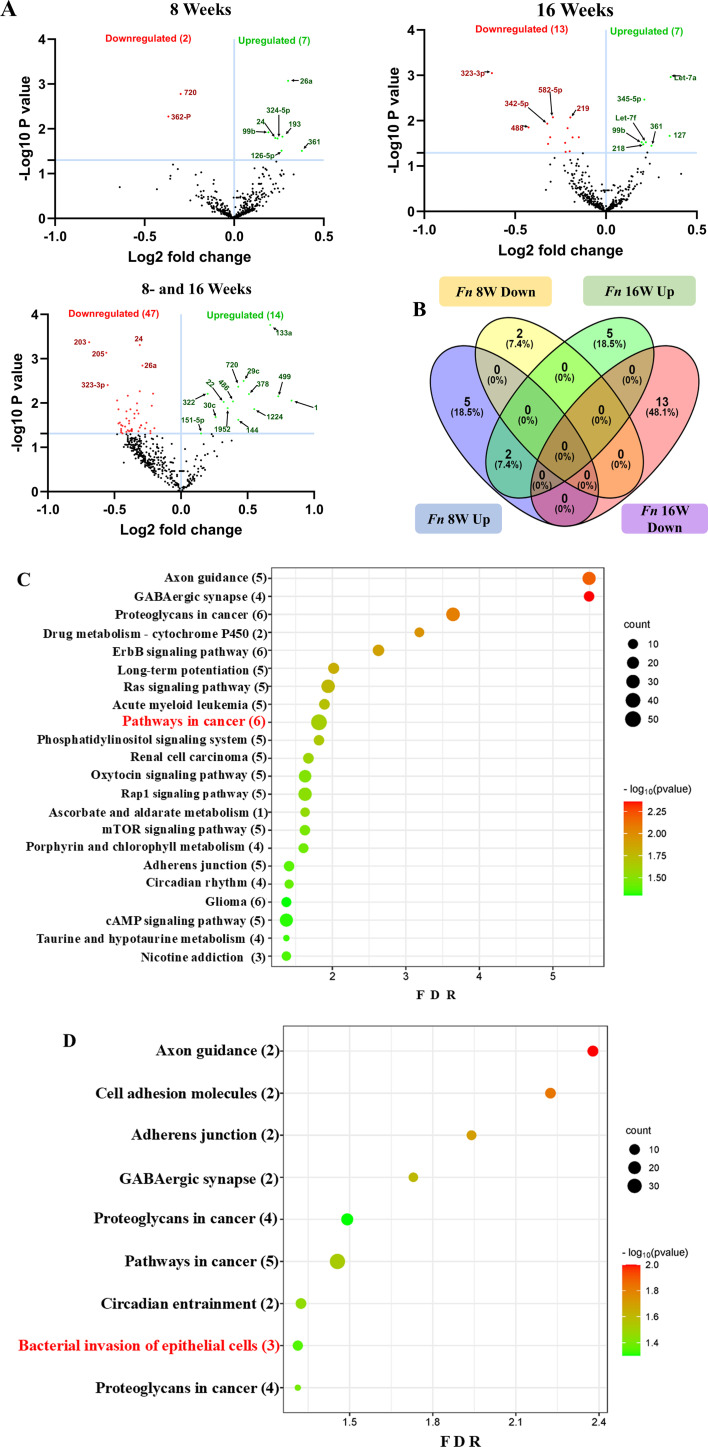
DE miRNAs in *F. nucleatum*-infected mandibles (8 and 16 weeks). (**A) **The volcano plot depicts the upregulated (green) and downregulated (red) miRNAs that showed a fold difference of ±1.1 with a *P*-value of <0.05. The log2 fold change is on the *x*-axis, and the negative log of the *P*-value is on the *y*-axis. The black dots stand for the miRNAs that do not pass the filter parameters. Seven significant upregulated miRNAs and two downregulated miRNAs were found in 8 weeks of *F. nucleatum*-infected mice compared to 8 weeks of sham-infected mice (*n* = 10). Seven significant upregulated miRs and 13 downregulated miRs were found in 16 weeks of *F. nucleatum*-infected mice compared to 16 weeks of sham-infected mice (*n* = 10). (**B)** Venn diagram analysis illustrates the distribution of DE miRNAs in 8 weeks and 16 weeks of infections with *F. nucleatum*. (C and D) Predicted functional pathway analysis of DE miRNAs from *F. nucleatum*-infected mandibles. Bubble plot of KEGG analysis on predicted target genes of DE miRNAs in *F. nucleatum*-infected mice at 16 weeks of infection compared to sham-infected mice. The KEGG pathways are displayed on the *y*-axis, and the *x*-axis represents the false discovery rate (FDR), which means the probability of false positives in all tests. The size and color of the dots represent the number of predicted genes and the corresponding *P*-value, respectively.

Two *F. nucleatum*-infected and two sham-infected groups were analyzed using high-throughput nCounter miRNA Expression Panels. To find the statistical significance in differentially expressed (DE)-miRNA, we performed volcano plot analysis. It was plotted against log2 fold change on the x-axis and the negative log of *P*-value on the y-axis. The identified downregulated miRNAs indicated in red and 14 upregulated miRNAs (green) showed a fold difference of +1.1 with a *P*-value of <0.05 in the 8 weeks of *F. nucleatum*-infected mice compared to the 16 weeks of infected group (Fig. 2A). All the black dots represent miRNAs that do not pass the filter parameters. In the 8 weeks of the infection study, seven miRNAs showed higher expression (e.g., miR-361-5p, miR-99b-5p) (Table 3). Similarly, seven miRNAs (e.g., miR-361-5p, miR-99b-5p) showed higher expression in 16 weeks of *F. nucleatum*-infected mandibles (Fig. 2B; Table 4).

Upregulated miRNAs reported in the 8 weeks have been associated as diagnostic miRNAs in humans (miR-361-5p), identified in the gingiva of PD patients (miR-26a-5p), attenuating the sepsis-induced myocardial injury in the mice (miR-193a-3p), associated with heart failure diagnosis in human subjects (miR-324-5p), regulating the cell survival in periodontal ligament cells (miR-24-3p), associated with mice PD infections induced by *T. denticola* (miR-126-5p) and *P. gingivalis* (miR-99b-5p). The 16 weeks of upregulated miRNAs were associated with PD diagnostic miRNA in humans (miR-361-5p), gingival tissue in chronic periodontitis patients (let-7a-5p), in the human periodontitis gingival tissue (let-7f-5p), reliable biomarker in patients with oral squamous cell carcinoma (miR-345-5p), clinical marker for atherosclerosis (miR-218-5p), and associated in mice PD infections induced by *P. gingivalis* (miR-99b-5p).

### DE miRNAs and functional pathway analysis

**Fig 3:**
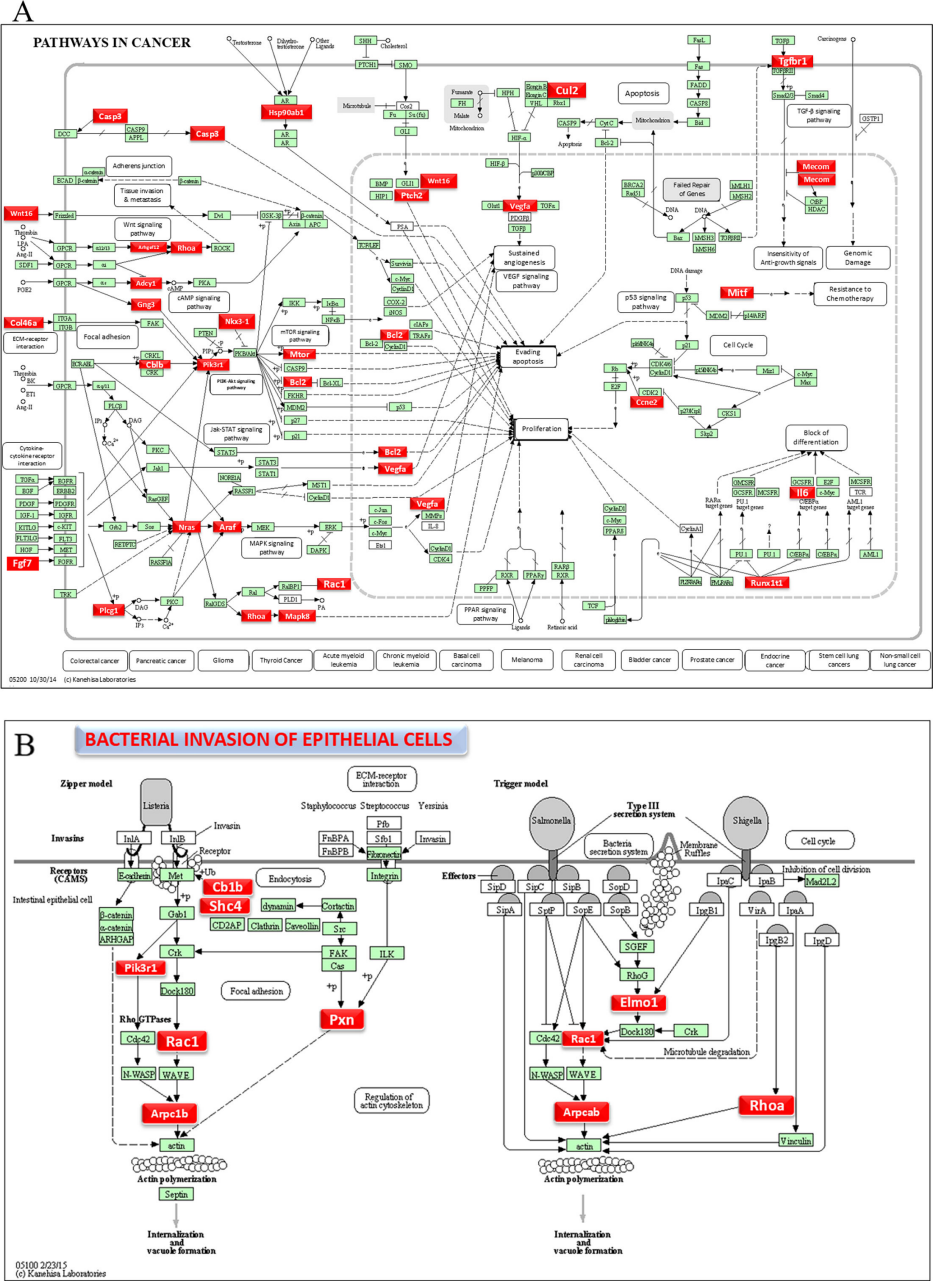
DE miRNAs and mirPath V.3—KEGG predicted functional pathways (KEGG pathway # mmu05200 and mmu05100). (A) A total of 37 genes (identified by KEGG) were involved in the pathways in cancer and (B) 10 genes were involved in the bacterial invasion of epithelial cells signaling pathway during 16 weeks of *F. nucleatum* infection. Red boxes indicate genes with significantly increased expression during pathways in cancer and bacterial invasion of epithelial cells. Green boxes indicate no change in gene expression. Many pathogenic bacteria can invade phagocytic and non-phagocytic cells and colonize them intracellularly, then become disseminated to other cells. Invasive bacteria induce their uptake by non-phagocytic host cells (e.g., epithelial cells) using two mechanisms referred to as the zipper model and trigger model. *Listeria, Staphylococcus, Streptococcus*, and *Yersinia* are examples of bacteria that can enter using the zipper model. These bacteria express proteins on their surfaces that interact with cellular receptors, starting signaling cascades that result in close apposition of the cellular membrane around the entering bacteria. *Shigella* and *Salmonella* are examples of bacteria entering cells using the trigger model. An arrow indicates a molecular interaction and a line without an arrowhead indicates a molecular interaction that results in inhibition.

**Fig 4:**
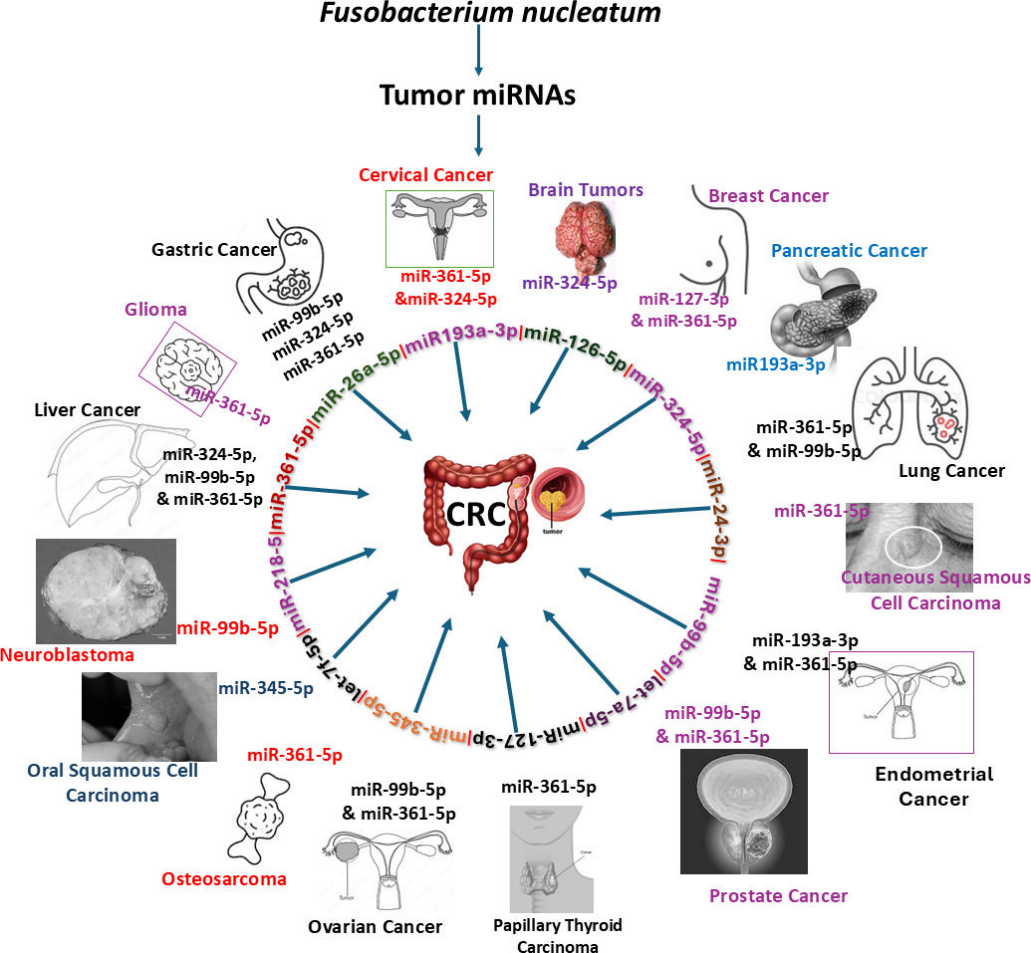
*F. nucleatum* upregulated miRNAs are linked with 13 multiple malignancies, including CRC. *F. nucleatum*-induced miR-361-5p alone is associated with 13 types of tumor, and 12 out of 14 miRNAs are associated with CRC.

Functional enrichment analysis for the upregulated and downregulated miRNAs was performed using DIANA-miRPath software to predict the biological function. The resulting KEGG pathway analysis revealed that the upregulated miRs in the 8-week analysis were associated with the pathways in cancer, axon guidance pathway, ErbR signaling pathway, renal cell carcinoma, mTOR signaling pathway, porphyrin metabolism, adherens junction, glioma, cAMP signaling pathway, etc. (Fig. 2C). The upregulated miRNAs in the 16-week analysis (miR-99b-5p, miR-361-5p, miR-345-5p, miR-218-5p, and miR-127-3p) associated with the proteoglycans in cancer, pathways in cancer, axon guidance pathway, cell adhesion molecules pathway, adherens junction pathway, GABAergic synapse pathway, circadian entrainment, bacterial invasion of epithelial cells, and proteoglycans in cancer (Fig. 2D). The miRs of miR-361-5p (*Cblb, Rhoa, Rac1*), miR-218-5p (*Pik3r1*, *Arpc1b, Elmo1, Shc4*), and miR-345-5p (*Pxn*) were associated with bacterial invasion of epithelial cells pathways having the target genes in the brackets (Fig. 3; Table S6). Pathways in cancer in both 8- and 16-week upregulated miRNAs have a single miRNA (miR-361-5p) that has the potential to interact with all of the genes in the pathway. The upregulated miRNAs associated with multiple malignancies including CRC are depicted in Fig. 4. The DE miRNAs upregulated in the 8 and 16 weeks of *F. nucleatum* infection have a regulatory role on 50 and 37 genes (Fig. 4), respectively, in the cancer pathways. The miR-361-5p, which is associated with pathways in cancer, has a regulatory target on 11 genes, including the *Crebbp* gene, which has the unusual largest interactions in the pathways of cancer. The reviewed information revealed that genetic aberrations of CREBBP/EP300 were observed in various types of solid tumors and hematologic malignancies and considered promising therapeutic targets (109). Detailed reported functions of the upregulated miRNAs during 8 and 16 weeks of *F. nucleatum* infection are shown in Tables 3 and 4, respectively. The list of upregulated miRNAs associated with the bacterial invasion of epithelial cells (Table S6), as well as pathways in cancers, is shown in Tables S7 and S8. The number of target genes for each upregulated miRNA in the 8- and 16-week- infection group was analyzed using miRTarBase. We used mmu-miR-361-5p as the example for an upregulated DE miRNA during 8 weeks of infection in identifying the target genes using miRTarBase (Table S9). Each miRNA has different target genes and a specific miRTarBase ID. *F. nucleatum*-infection-induced DE-upregulated mmu-miR-361-5p has 15 different target genes with 15 different miRTarBase IDs as stated in Table S9. The list of target genes for 16 weeks of upregulated miRNAs and the miRTarBase IDs are shown in Table S10.

### Machine learning analysis of NanoString miRNA copies

**Fig 5:**
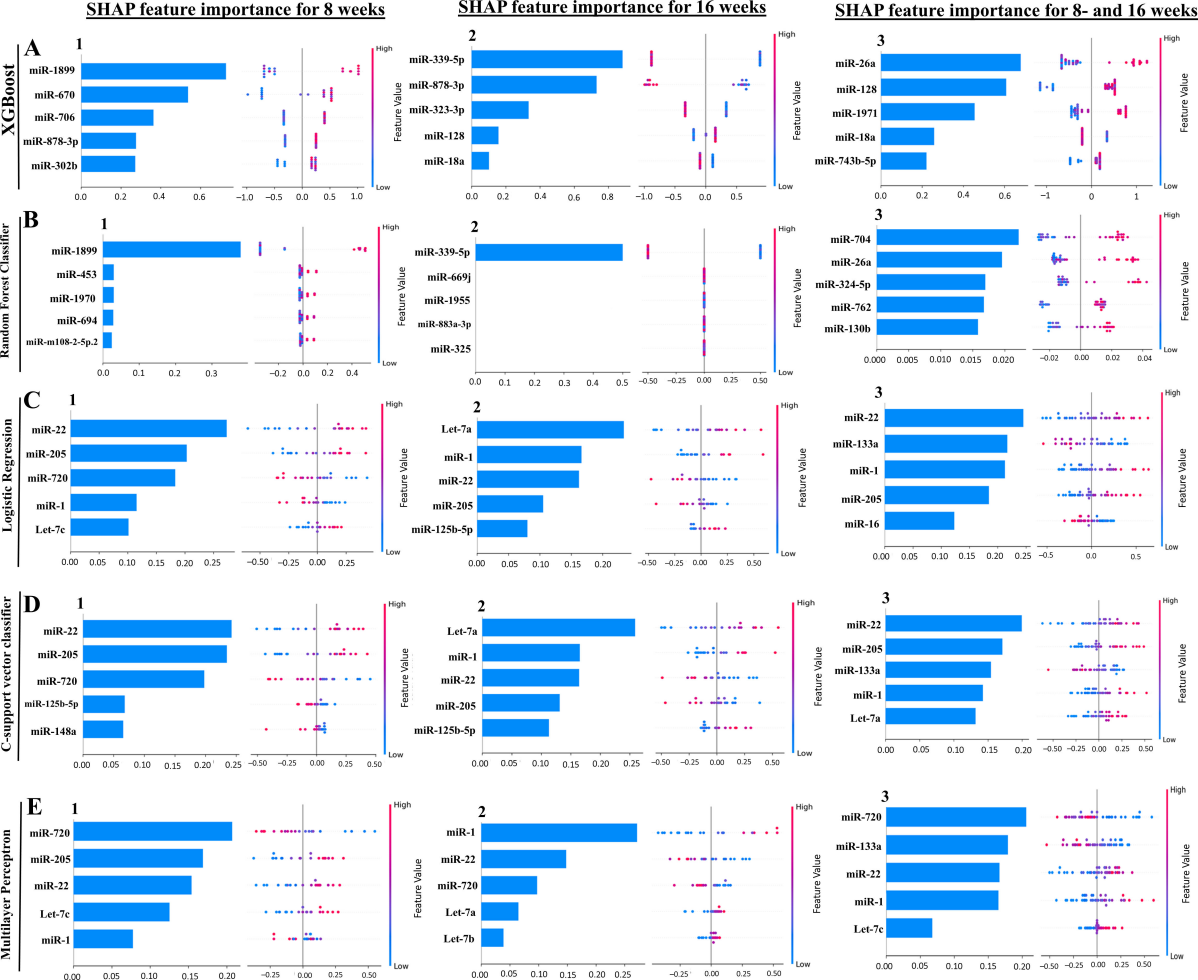
A summary of the most prominent features in the five machine learning models using SHapley Additive exPlanations (SHAP) values. In panels A–E, feature importance is ranked from the top (the most important) to the bottom (the least important). The *x*-axis shows the impact that a feature has on the model. The bar charts show the overall impact of a feature, whereas the swarm plot shows both the positive and negative impacts. In the swarm plots, each dot represents an instance of a miRNA variable, and the color bar shows the variable’s value (high to low). The three study groups are (1) mice assessed at 8 weeks, (2) mice assessed at 16 weeks, and (3) 8 and 16 weeks together.

Our ML analysis results are divided into two groups: tree-based methods, consisting of XGB and RFC, and non-tree-based methods, consisting of LR, SVC, and MLP. In the 8-week data set, mmu-miR-1899 had the highest impact on the tree-based models (Fig. 5A1, XGB; Fig. 5B1, RFC), LR and SCV were most impacted by mmu-miR-22, and miR-720 in MLP had the highest impact (Fig. 5C1, LR; Fig. 5D1, SVC; Fig. 5E1, MLP). For the 16-week data set, mmu-miR-339-5p had the highest impact on the tree-based models (XGB, RFC; Fig. 5A2 and B2), while mmu-miR-7a had the highest impact on LR and SVC, and miR-1 in MLP was most impacted by miR-1 (LR, SVC, Fig. 5C2 and D2 and MLP; Fig. 5E2). Finally, in the combined 8- and 16-week data set, the most impactful miRNAs were mmu-miR-26a-5p in XGB, miR-704 in RFC was again the most impactful for the tree-based models (XGB, RFC; Fig. 5A3 and B3), mmu-miR-22 in LR and SVC, and mmu-miR-720 in MLP (LR, SVC, Fig. 5C3 and D3 and MLP, Fig. 5E3). The results of the SHAP value analysis are summarized in Fig. S1 to S5; Table 5 summarizes the most impactful miRNAs for each ML model and descriptions of the miRNA functions, and Table 6 summarizes the top five important miRNAs.

**TABLE 5:** Summary of the miRNA and its importance for each machine learning model^*a*^

miRNA (feature rank)	ML model	MIMAT#	Target functions
8-week analysis			
mirR-22 (1)	LR, SVC	MIMAT0000531	Upregulated in periodontal disease and obesity (110).
miR-205 (2)	LR, SVC, MLP	MIMAT0000238	Downregulated in chronic periodontitis patients (111, 112).
miR-720 (3)	LR, SVC	MIMAT0003484	Novel miRNA regulating the differentiation of dental pulp cells (113).
miR-1899 (1)	XGB, RFC	MIMAT0007869	–^*b*^
16-week analysis			
let-7a-5p (1)	LR, SVC	MIMAT0000521	Downregulated in aggressive periodontitis patients (88). Promotes the osteogenesis of bone marrow mesenchymal stem cells (114).
miR-1(2)	LR, SVC	MIMAT0000123	Noninvasive biomarker for breast cancer (115). Upregulated in patients with myocardial infarction (116).
miR-22 (3)	LR, SVC	MIMAT0000531	Shown in the 8-week analysis
miR-205 (4)	LR, SVC	MIMAT0000238	Shown in the 8-week analysis
miR-125b-5p (5)	LR, SVC	MIMAT0000135	Associated with osteogenic differentiation (117). Overlapping miRNA between periodontitis and nonalcoholic fatty liver disease (118).
miR-339-5p (1)	XGB, RFC	MIMAT0000584	Most predictive periodontal miRNA in the *T. forsythia-*induced mice periodontitis (33).
8- and16-week analysis			
miR-22 (1)	LR, SVC	MIMAT0000531	Shown in the 8-week analysis
miR-133a (2)	LR, MLP	MIMAT0000145	Upregulated in the *T. denticola-*induced mice periodontitis (32).
miR-1 (4)	SVC, MLP	MIMAT0000123	Shown in the 16-week analysis

^*a*^ The rank (or importance) of miRNA in predicting whether the mouse was infected is in parentheses. The higher the rank, the more important the miRNA was for making the prediction. For the 8- and 16-week cohort, XGB and RFC did not agree on the importance of miRNA. Models: logistic regression (LR), C-support vector classifier (SVC), multilayer perceptron (MLP), random forest classifier (RFC), and extreme gradient boosting (XGB).

^*b*^ “–” indicates that no research articles were found related to that microRNA.

**TABLE 6:** Summary of the importance and miRNA for each machine learning model^*a*^

Cohort	miRNA	miRNA feature rank				
		1	2	3	4	5
8 weeks	miR-22	LR, SVC	–^*b*^	MLP	–	–
	miR-205	–	LR, SVC, MLP	–	–	–
	miR-720	MLP	–	LR, SVC	–	–
	miR-1	–	–	–	LR	MLP
	let-7c	–	–	–	MLP	LR
	miR-125b-5p	–	–	–	SVC	–
	miR-148a	–	–	–	–	SVC
	miR-1899	XGB, RFC	–	–	–	–
	miR-670	–	XGB	–	–	–
	miR-706	–	–	XGB	–	–
	miR-878-3p	–	–	–	XGB	–
	miR-302b	–	–	–	–	XGB
	miR-453	–	RFC	–	–	–
	miR-1970	–	–	RFC	–	–
	miR-694	–	–	–	RFC	–
	miR-m108-2-5p.2	–	–	–	–	RFC
16 weeks	let-7a-5p	LR, SVC	–	–	MLP	–
	miR-1	MLP	LR, SVC	–	–	–
	miR-22	–	MLP	LR, SVC	–	–
	miR-205	–	–	–	LR, SVC	–
	miR-125b-5p	–	–	–	–	LR, SVC
	miR-720	–	–	MLP	–	–
	let-7b	–	–	–	–	MLP
	miR-339-5p	XGB, RFC	–	–	–	–
	miR-878-3p	–	XGB	–	–	–
	miR-323-3p	–	–	XGB	–	–
	miR-128	–	–	–	XGB	–
	miR-18a	–	–	–	–	XGB
8 and 16 weeks	miR-22	LR, SVC	–	MLP	–	–
	miR-133a	–	LR, MLP	SVC	–	–
	miR-1	–	–	LR	SVC, MLP	–
	miR-205	–	SVC	–	LR	–
	miR-16	–	–	–	–	LR
	miR-720	MLP	–	–	–	–
	let-7a-5p	–	–	–	–	SVC
	let-7c	–	–	–	–	MLP
	miR-26a-5p	XGB	RFC	–	–	–
	miR-128	–	XGB	–	–	–
	miR-1971	–	–	XGB	–	–
	miR-18a	–	–	–	XGB	–
	miR-743b-5p	–	–	–	–	XGB
	miR-704	RFC	–	–	–	–
	miR-324-5p	–	–	RFC	–	–
	miR-762	–	–	–	RFC	–
	miR-130b	–	–	–	–	RFC

^*a*^ The higher the rank, the more important the model found the miRNA to be for predicting if the mouse was infected. Models: logistic regression (LR), C-support vector classifier (SVC), multilayer perceptron (MLP), random forest classifier (RFC), and extreme gradient boosting (XGB). LR—logistic regression, SVC-C—support vector classifier, MLP—multilayer perceptron, RFC—random forest classifier, and XGB—extreme gradient boosting.

^*b*^ “–” indicates that no machine language model ranked the miRNA at the that specific rank.

In addition to determining which miRNAs were most impactful on the ML models (Table 5), we also found high levels of agreement in the miRNAs ranked 2 to 5 (Table 6). Interestingly, the tree-based models did not agree on the importance of miRNAs for ranks between 2 and 5 in the 8 weeks, 16 weeks, and the combined 8 and 16 weeks of data sets. In the 8 weeks of data set, the non-tree-based models ranked miR-205 as the second most important feature, and LR and SVC ranked miR-720 third. In the 16 weeks of data set, LR and SVC ranked miR-1 as second, miR-22 as third, miR-205 as fourth, and miR-125b-5p as the fifth most important feature. Lastly, in the combined 8 and 16 weeks of data set, LR and MLP ranked miR-133a as the second most important feature, and SVC and MLP ranked miR-1 as fourth.

## DISCUSSION

The frequent involvement of *F. nucleatum* in extra-oral systemic infections and comorbidities (2), including adverse pregnancy outcomes, and various malignancies such as CRC (14, 15) supports this species’s role in multiple disease pathogeneses. Since *F. nucleatum* can survive, spread hematogenously, and replicate at sites distant from the oral cavity, it uses its FadA adhesion to bind and invade both endothelial and epithelial cells (119), which plays a significant role in the development of periodontitis and its systemic comorbidities. Although the etiologic role of *F. nucleatum* as an important bacterium in the progression of PD is known, the underlying molecular genetic mechanisms are not completely understood. Recently, several studies have suggested that aberrant expression of miRNAs is involved in both infectious and non-infectious diseases, influencing the initiation and progression of pathology and the development of miRNAs as diagnostic biomarkers in CRC. To the best of our knowledge, this study will be the first investigation to examine *F. nucleatum* oral infection, global miRNA induction, and genes involved in periodontitis.

In this report, we focused on how *F. nucleatum* selectively modulates host gingival epithelial cell responses with robust and specific miRNA expression during the progression of PD. We demonstrate that chronic oral infection with *F. nucleatum* in male and female mice results in physiological colonization/infection of the gingival surfaces after four infection cycles, invades gingival epithelium, and induces PD outcome measures such as ABR, which are significantly higher in mice at both 8 and 16 weeks of infection time points. In addition, *F. nucleatum* can spread via intravascular dissemination to distal organs, including the heart, suggesting its invasive potential and ability to modulate the host immune response. This invasive potential to the heart and other tissues was robust when *F. nucleatum* was administered with late colonizers such as *P. gingivalis*, *T. denticola*, and *T. forsythia* and the early colonizer *S. gordonii* (34), suggesting their physiological, nutritional, and metabolic synergistic interactions in the gingiva. This co-dependence enhances microbial multiplication and invasion of the gingival epithelium, leading to systemic intravascular dissemination. The partial human mouth microbes (PHAMM) ecological time sequential polybacterial periodontal infection (ETSPPI) model involves an array of five bacteria-mediated intraoral infections, which colonize bacteria on the gingiva, induce periodontitis, and lead to induced sex-specific miRNA expression. These miRNAs are linked to numerous systemic diseases and comorbidities (34). Monobacterial intraoral infections with *P. gingivalis, T. denticola, T. forsythia,* and *S. gordonii* also induced periodontitis with robust alterations in miRNAs in mice models (31–33, 37).

This study analyzed 577 mouse miRNAs in mandibles from *F. nucleatum*-infected and sham-infected mice using the high-throughput NanoString nCounter miRNA profiling. Most of the DE miRNAs were unique, except for two miRNAs (361, 99b), and specific to the time point, indicating that miRNA induction is transient and time-dependent. A total of 29 miRNAs were DE in mandible tissue during 8 and 16 weeks of *F. nucleatum* infection compared with sham infection. Of these, 14 miRNAs were upregulated, and 15 miRNAs were downregulated. Among all the 14 upregulated miRNAs at 8 and 16 weeks, four miRNAs were reported in human periodontitis studies, indicating that these upregulated miRNAs are associated with the induction of PD. Specifically, miR-26a-5p was downregulated in the gingiva of periodontitis patients (55), mmu-let-7a-5p was found in the saliva of patients with aggressive periodontitis (88), and in the gingival tissue of chronic periodontitis patients (89), and mmu-let-7f-5p was found in human periodontitis gingival tissue (66), indicating that preclinical *in vivo* miRNA data corroborate with clinical PD miRNA data. Three of the upregulated miRNAs have been associated with preclinical mouse studies: miR-99b-5p in *P. gingivalis*-induced PD (31), miR-126-5p in *T. denticola*-induced PD (32), and miR-127-3p in *T. forsythia*-induced PD (33), and were also expressed during *F. nucleatum* infection. Furthermore, the two miRNAs, miR-361-5p and miR-99b-5p, were commonly expressed and upregulated at both time points.

In addition, the miRNAs miR-361-5p, miR-193a-3p, miR-324-5p, miR-99, miR-127-3p, miR-345-5p, miR-218-5p, and let-7f-5p are associated with cardiovascular diseases (54, 75, 95, 106, 120–122). miR-324-5p and miR-345-5p are linked to adipocyte differentiation (123, 124), while miR-127-3p, miR-345-5p, and miR-99 are associated with tumor malignancies (96, 98, 125–127). Interestingly, a single miRNA can be associated with different disease conditions. For example, miR-324-5p is linked to osteoporosis (76) and multiple myeloma (128), let-7a-5p is associated with various inflammatory conditions (90, 129), chemotherapy-exposed mouse ovaries (130), an antifibrotic role under hypoxic stress (131), and asthma (91). miR-24-3p is involved in phagocytosis (77), periodontal ligament cells (132), and gingival fibroblasts (104), let-7f-5p is linked to abdominal aortic aneurysm (102) and lupus nephritis (103). Different miRNAs are also associated with microbial infection-exposed immune cells. For example, miR-99 is linked to *M. tuberculosis*-infected murine dendritic cells (79), miR-127-3p is involved in macrophage anti-microbial responses (133), and miR-24-3p is associated with *S. aureus*-caused osteomyelitis (134).

Recent reports demonstrate microbiological, molecular, and genetic links between *F. nucleatum* and multiple digestive tract cancers, including esophageal, gastric, pancreatic, and CRC. Interestingly, our study showed 37 significantly increased gene expressions in multiple cancers and several miRNAs associated with both multiple cancers, including CRC and PD in humans (135), some of which also link PD with inflammatory systemic comorbidities. Several studies identified oncogenic marker miRNAs (oncomiRs) in CRC tumors (miR-126-5p, miR-99b-5p, miR-26a-5p, miR-24-3p, miR-361-5p, miR-193a-3p, miR-218-5p, let-7a-5p, and let-7f-5p), which were also expressed in mouse mandibles during *F. nucleatum* infection. This indicates a link between invasive *F. nucleatum* and multiple tumors, including CRC, further strengthening the association between periodontitis and CRC. By contrast, *P. gingivalis* oral infection in mice showed DE miR-185, miR-22, miR-152, miR-423, miR-151, miR-28, and miR-145, which were also found to be upregulated in various malignancies such as prostate, breast, glioma, gastric, and hepatocellular carcinoma (31). The data indicate that periodontal bacteria-expressed miRNA in gingival tissues and other systemic diseases have common mRNA targets mediating disease progression.

KEGG pathway analysis revealed that the most target genes of upregulated miRNAs during the 8 week period were linked with several pathways, including those involved in cancer, axon guidance, GABAergic synapse, proteoglycans in cancer, drug metabolism-cytochrome P450, ErbR signaling pathway, acute myeloid leukemia, phosphatidylinositol signaling system, renal cell carcinoma, adherens junction, glioma, and cAMP signaling pathway.

Periodontal disease-causing infectious oral microbes are commonly detected in atherosclerotic plaques and are associated with many systemic diseases. *F. nucleatum* is linked with more systemic diseases than other known periodontal microbes and has been isolated from more than 10 sites of systemic infection (2). Periodontal infection is associated with atherosclerosis, where atherosclerotic arteries of patients tested positive for *F. nucleatum* genomic DNA (84%) (136). There is evidence that *F. nucleatum* sub-species have fine-tuning capabilities to drive disease progression and niche colonization in the disease habitat. Zepeda-Rivera et al. revealed that the *F. nucleatum* strains causing colorectal cancer (*Fna* C2 strains) have a close genome sequence with *F. nucleatum* present in the oral cavity of patients with colorectal cancer (135). Polymicrobial oral infection in the mice, including *F. nucleatum* as a member, caused the hematogenous dissemination of *F. nucleatum* organisms into the heart and aorta (137). In rodent models, the predicted hypothesis of transient bacteremia caused by *F. nucleatum* facilitated its transmission from the oral cavity to the uterus, resulting in premature delivery, stillbirths, and no sustained live births (138). The abundance/enrichment of *F. nucleatum* in colorectal tumor specimens confirmed its correlation with colorectal cancer (139). One of the limitations of the current study is the inability to utilize saliva in this microRNA analysis.

The machine learning model analysis for *F. nucleatum* showed some similarities with the ML algorithm in the *S. gordonii* (*Sg*) monoinfection. In our present study, tree-based models identified miR-339-5p and miR-323-3p as significant features, which were also observed in *S. gordonii-*induced PD mice. The miRNA features miR-22, miR-205, miR-720, miR-1, mmu-let-7c, and mmu-miR-7a were identified in non-tree-based models in both the *S. gordonii* study and our present study. The miR-125b-5p feature was uniquely observed in the MLP model of *S. gordonii* infection and the LR and SVC models of our present study. Similarly, miR-133a was an observed feature in the SVC model of the *S. gordonii* PD studies and was reported in the non-tree-based models of our present study (37). In addition, tree-based models in our present study and in *T. forsythia*-induced PD mice identified common features including miR-18a, miR-339-5p, miR-130b, and miR-704 (33). We acknowledge that further research is necessary to elucidate how *F. nucleatum* influences the development of PD versus cancer. Furthermore, we recognize that key factors may still be unidentified that direct the progression toward PD rather than cancer, despite the robust induction of the many oncogenic miRNAs. This area requires more investigation to fully understand the underlying mechanisms and potential therapeutic targets.

In conclusion, this is the first *in vivo* study using rodent models of *F. nucleatum* intraoral infection-induced periodontitis with emphasis on global miRNA profiling. Elevated miR-361 expression during 8 and 16 weeks of infection was linked to multiple malignancies. miR-127 and miR-26a might be involved in the initial immune response against *F. nucleatum* infection. miR-361 and miR-99b were unique in both 8 and 16 weeks of *F. nucleatum* infection. It is interesting that miR-126-5p has been reported as a potential biomarker in patients with periodontitis and coronary artery disease. These findings highlight *F. nucleatum’s* multi-pathogenic role in periodontitis and systemic comorbidities, and specifically, 13 oncogenic miRNAs (oncomiRs) are linked with CRC. Further studies should focus on the role of miR-361 in periodontitis and as therapeutic miRNA in periodontitis.

## Data Availability

The data that support the findings of this study are openly available at https://www.ncbi.nlm.nih.gov/geo/query/acc.cgi?acc=GSM7915885 (accessed on 4 December 2023).

## SUPPLEMENTAL MATERIAL

Supplemental material msystems.01732-24-s0001.docx Supplemental figures and tables.

ASM does not own the copyrights to Supplemental Material that may be linked to, or accessed through, an article. The authors have granted ASM a non-exclusive, world-wide license to publish the Supplemental Material files. Please contact the corresponding author directly for reuse.
