# Supplementary material for: Exploring the complex interplay between oral Fusobacterium nucleatum infection, periodontitis, and robust microRNA induction, including multiple known oncogenic miRNAs
Source: mSystems. 2025 Jun 25;10(7):e01732-24. doi: 10.1128/msystems.01732-24 (PMC12282186; doi:10.1128/msystems.01732-24)
Supplement: Supplemental material — Supplemental figures and tables. [file msystems.01732-24-s0001.docx]

Article

**Exploring the complex interplay between oral *Fusobacterium nucleatum* infection, periodontitis, and robust microRNA induction including multiple known oncogenic miRNAs.**

Syam Jeepipalli ^1†^, Aravindraja C ^1,†‡^, William Duncan ^2^, V. M. Krishna ^1^, Bikash Sahay ^3^, Edward K. L. Chan ^4^, and L. Kesavalu ^1,4*^.

^1^ Department of Periodontology, College of Dentistry, University of Florida, Gainesville, FL 32610, USA; aravindrchairman@ufl.edu (C.A.); sjeepipalli@dental.ufl.edu (S.J.); kvekariya@ufl.edu (K.M.V.)

^2^ Department of Community Dentistry and Behavioral Science, College of Dentistry, University of Florida, Gainesville, FL 32610, USA; duncanw@ufl.edu

^3^ Department of Infectious Diseases and Immunology, College of Veterinary Medicine, University of Florida, Gainesville, FL 32610, USA; sahayb@ufl.edu

^4^ Department of Oral Biology, College of Dentistry, University of Florida, Gainesville, FL 32610, USA; echan@ufl.edu

*****Correspondence: kesavalu@dental.ufl.edu; Tel.: +1-352-273-6500

† These authors contributed equally to this work.

‡ Current address: Department of Neurology, College of Medicine, University of Florida,

Gainesville, FL 32610, USA.

**Supplementary Material**

**
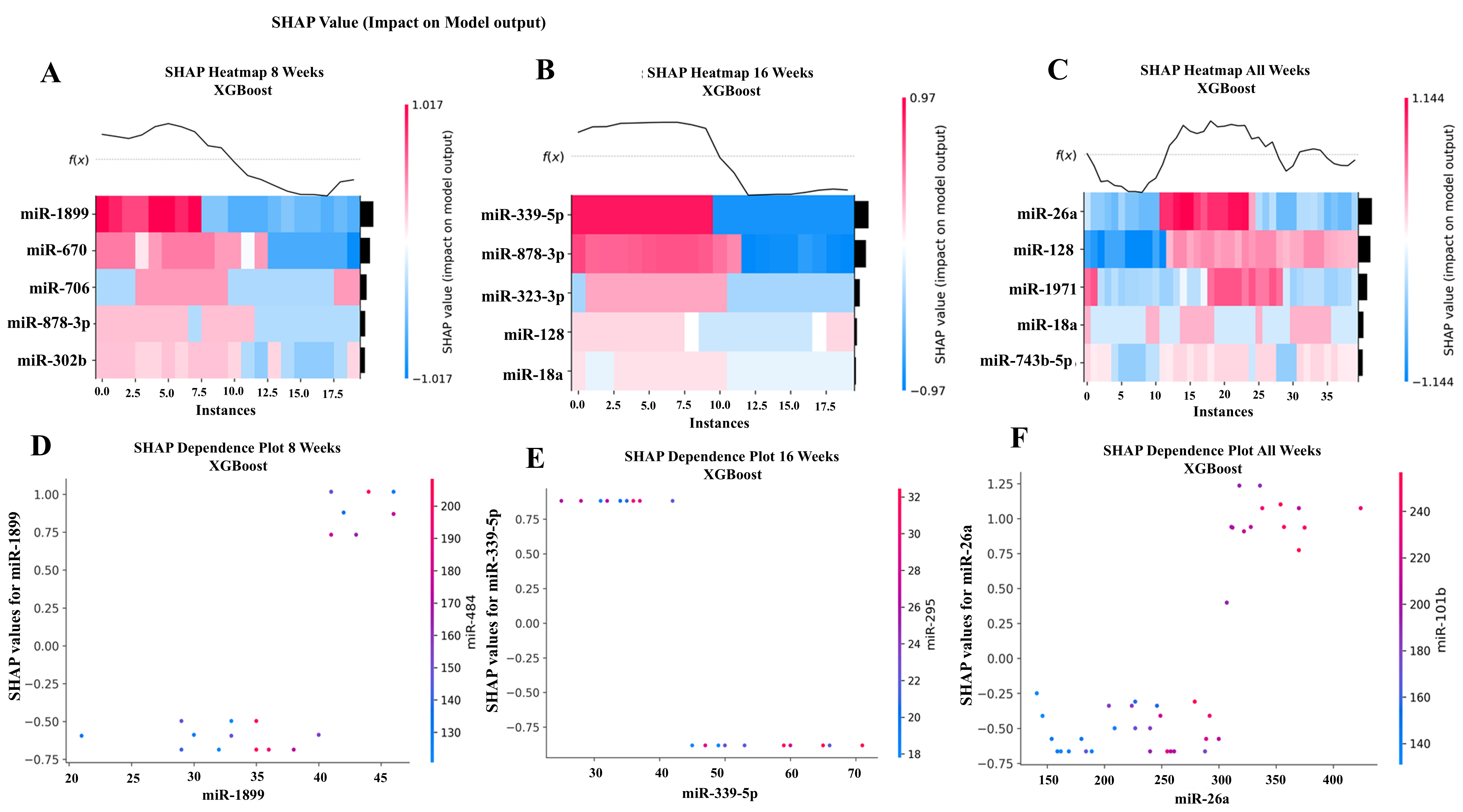
**

**SUPPLEMENTARY FIGURE S1** A summary of the most important features in the XGBoost model using SHAP values. In figures (A–C), feature importance is ranked from the top (the most important) to the bottom (the least important). (A–C), the x-axis represents each mouse (instance) in the cohort, and the y-axis is the feature ranking. The color of the cell shows the amount of impact (i.e., the SHAP value) that particular feature inflicted on that feature. The topmost section (i.e., f(x)) of the heatmap shows the predicted infection status for each instance. In all three cohorts, there is a strong correlation between high values for the topmost feature and the model predicting that the mouse was infected. In (D–F), the relation between the topmost feature and the feature it most depends on is shown. Each dot represents a mouse and the x axis shows the value of the miRNA variable. The left y-axis shows the impact (i.e., the SHAP value) the x-axis variable has on the mode. The right y-axis shows the value of the variable that the x-axis variable interacts with.

**
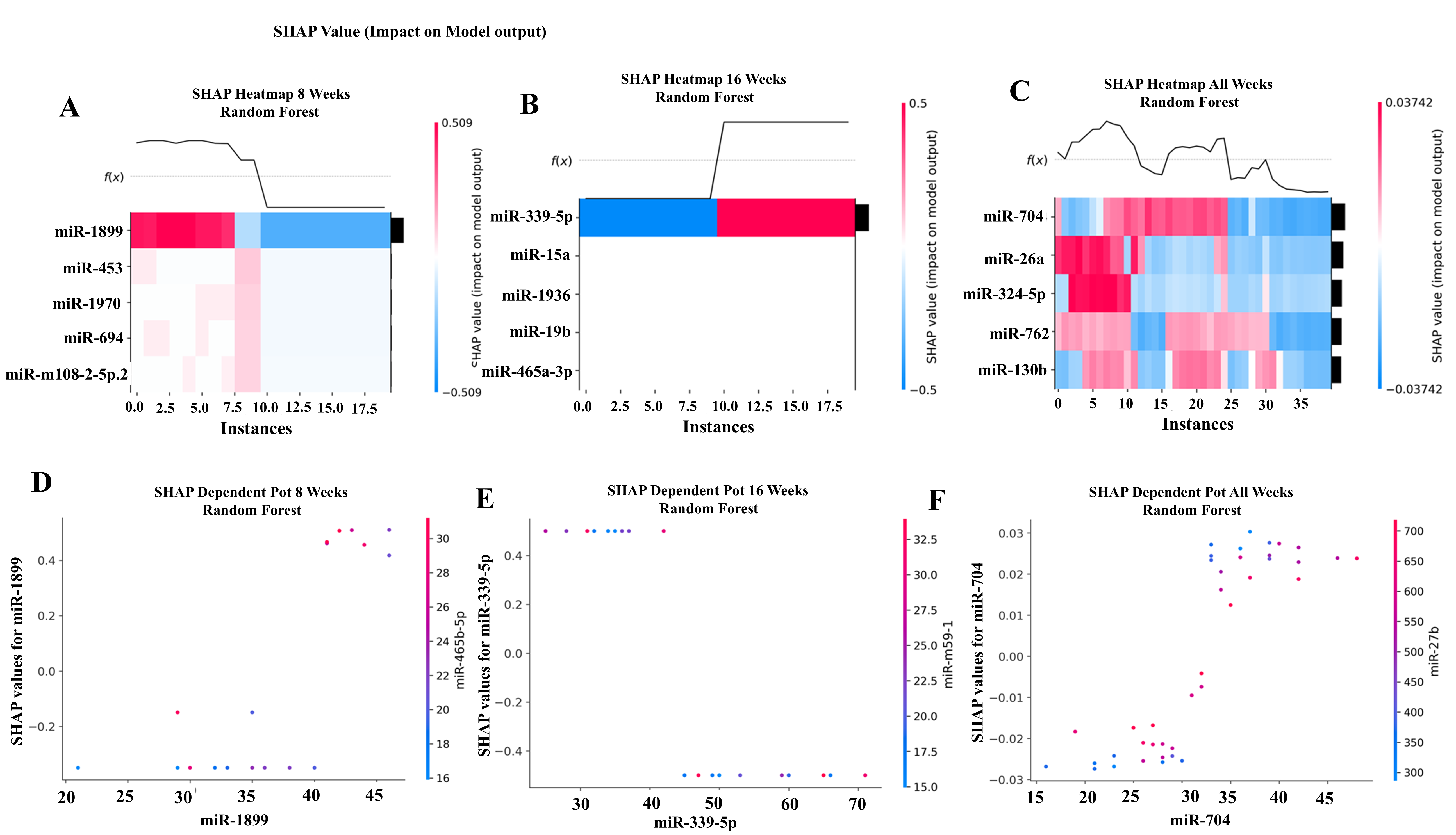
**

**SUPPLEMENTARY FIGURE S2** A summary of the most important features in the Random Forest Classifier (RFC) model using SHAP values. See the description in Figure 5 for an explanation about the graph plots.

**
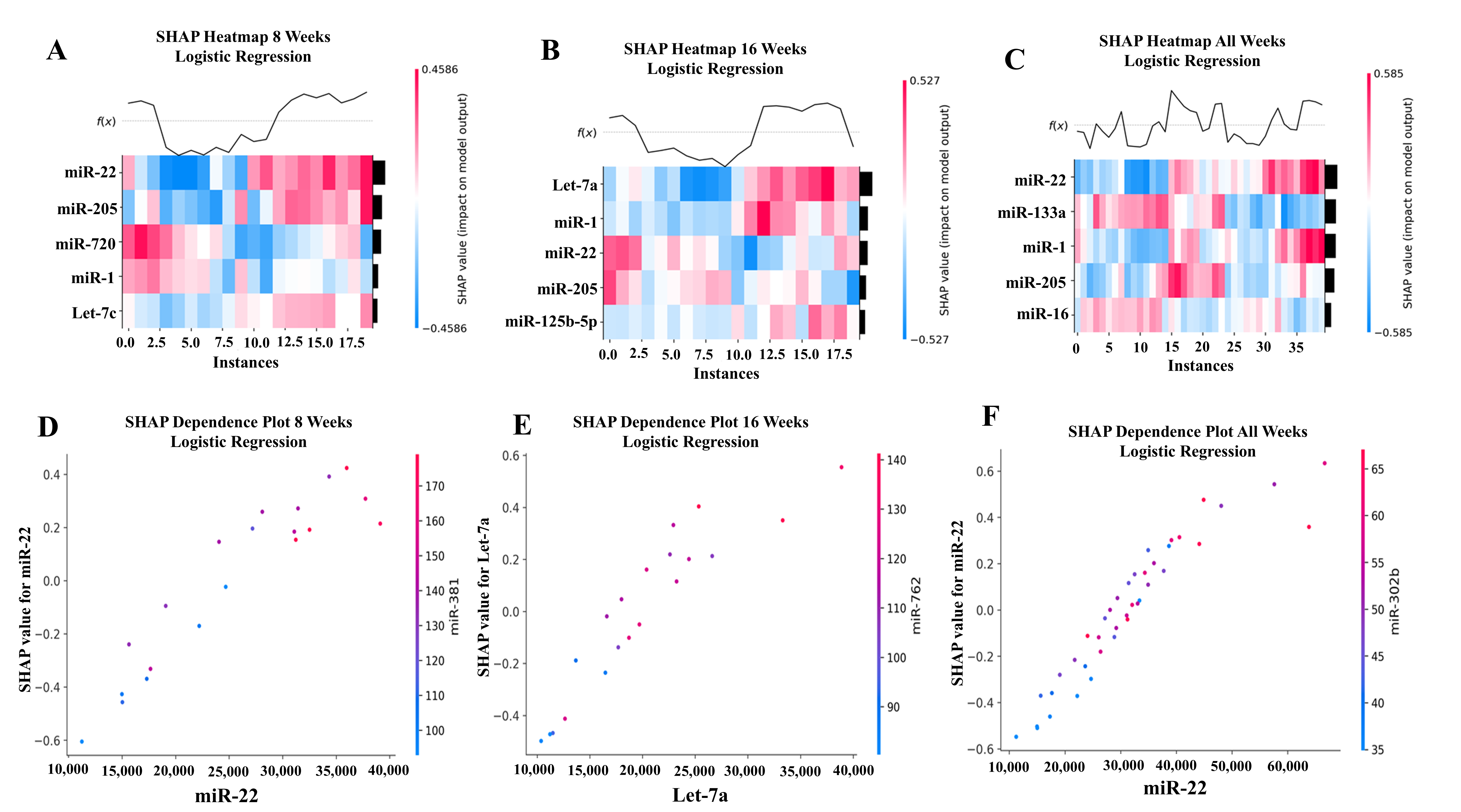
**

**SUPPLEMENTARY FIGURE S3** A summary of the most important features in the Logistic Regression (LR) model using SHAP values. See the description in Figure 5 for an explanation about the graph plots.


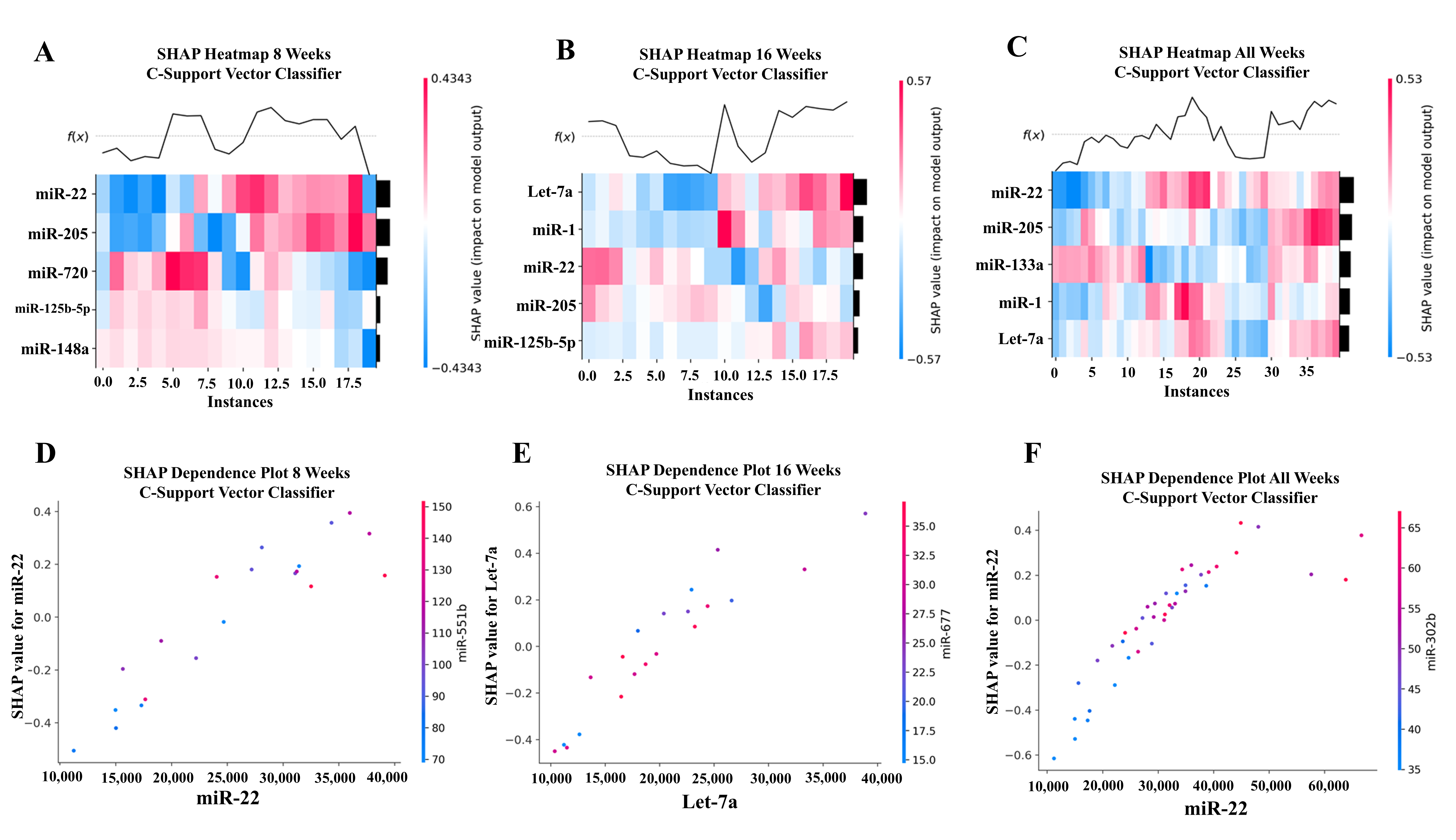


**SUPPLEMENTARY FIGURE S4** A summary of the most important features in the C-Support Vector Classifier (CSV) model using SHAP values. . See the description in Figure 5 for an explanation about the graph plots.


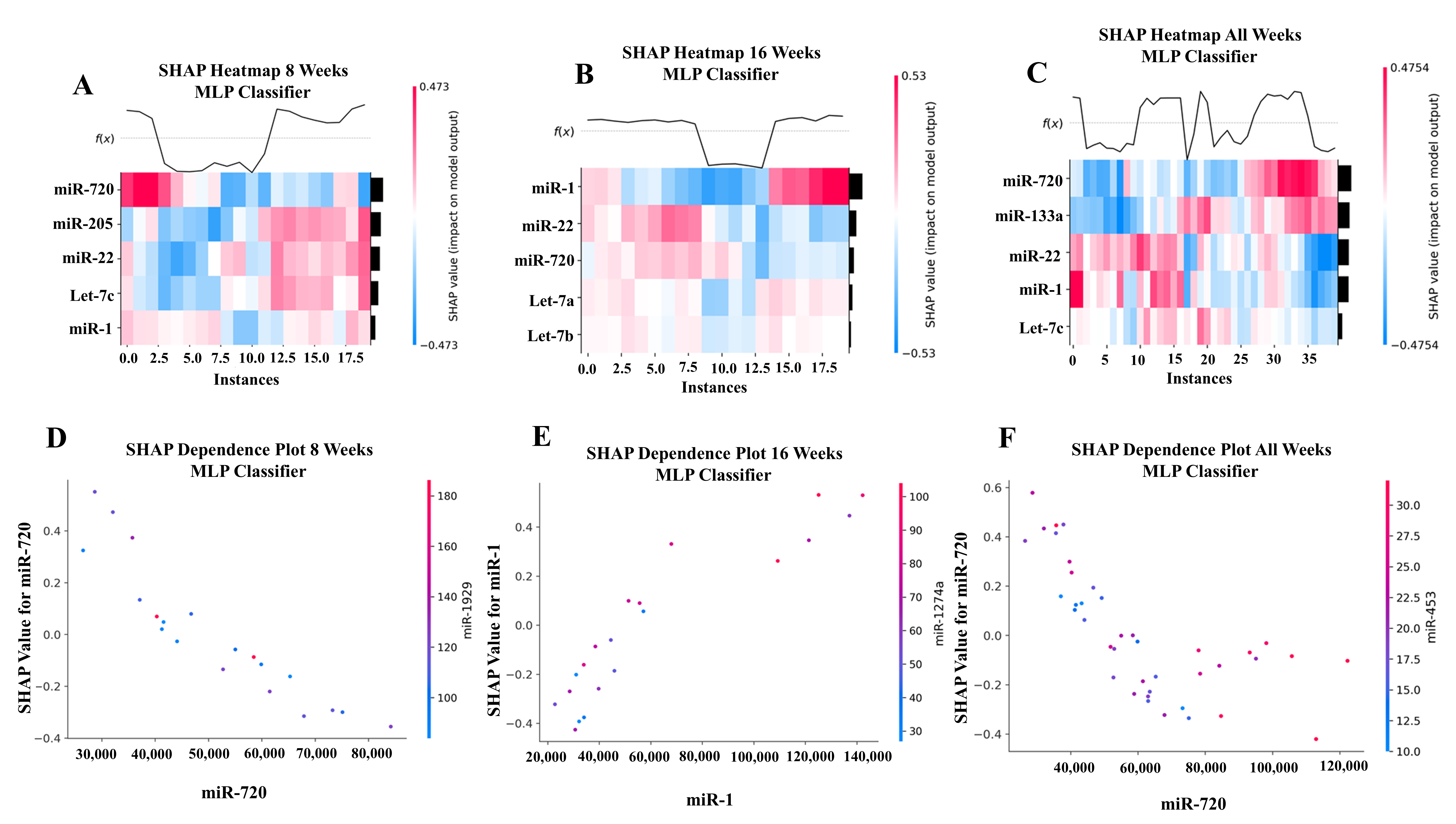


**SUPPLEMENTARY FIGURE S5** A summary of the most important features in the Multilayer Perceptron (Neural Network) model using SHAP values. See the description in Figure 5 for an explanation about the graph plots.

**TABLE S1** *F. nucleatum* PCR test for Bacterial dissemination to distal organs.

| **Group/Bacteria/Infection (Weeks) PCR Positive samples (n=10)** | | | | | | | |
| --- | --- | --- | --- | --- | --- | --- | --- |
|  |  | Heart | Liver | Lungs | Kidney | Spleen | Brain |
| Group I/*F. nucleatum* [8 weeks] |  | 3 | 1 | 2 | 0 | 0 | 0 |
| Group II/Sham infection [8 weeks] |  | 0 | 0 | 0 | 0 | 0 | 0 |
| Group III/*F. nucleatum* [16 weeks] |  | 5 | 0 | 6 | 1 | 0 | 0 |
| Group IV/Sham infection [16 weeks] |  | 0 | 0 | 0 | 0 | 0 | 0 |

To analyze the systemic bacterial infection, total genomic DNA from an aliquot of the mouse heart (comprising the right atrium and right ventricle), lungs, kidney, and liver was extracted. The extracted genomic DNA was examined for the presence of *Fn*-bacterial DNA specific 16S rRNA gene primers. The dissemination of bacteria from the oral gingival surface to the heart represents *F. nucleatum* invasive potential.

**TABLE S2** *F. nucleatum* 8 weeks infection and 16 weeks infection-induced downregulated miRNAs.

| **miRNAs in 8 weeks of infection** | | |
| --- | --- | --- |
| miRNAs | Fold change | p-value |
| miR-362-3p | -1.29 | 0.0053 |
| miR-720 | -1.23 | 0.0017 |
| **miRNAs in 16 weeks of infection** | | |
| miR-323-3p | -1.55 | 0.0009 |
| miR-488 | -1.34 | 0.0141 |
| miR-342-5p | -1.25 | 0.0116 |
| miR-376c | -1.25 | 0.0326 |
| miR-1274a | -1.24 | 0.0232 |
| miR-582-5p | -1.22 | 0.0084 |
| miR-151-3p | -1.17 | 0.0309 |
| miR-339-5p | -1.17 | 0.0487 |
| miR-27a | -1.16 | 0.0146 |
| miR-219 | -1.15 | 0.0085 |
| miR-511 | -1.15 | 0.0474 |
| miR-302b | -1.14 | 0.0233 |
| miR-350 | -1.11 | 0.0231 |

**TABLE S3** *F. nucleatum* infection-induced upregulated and downregulated miRNAs in 8 weeks infection Vs 16 weeks infection.

| **Upregulated miRNAs** | | | | | | | |
| --- | --- | --- | --- | --- | --- | --- | --- |
| **S. No** | **miRNAs** | **Fold change** | **p-value** | **S. No** | **miRNAs** | **Fold change** | **p-value** |
| 1 | miR-1 | 1.78 | 0.0088 | 8 | miR-144 | 1.35 | 0.0239 |
| 2 | miR-499 | 1.66 | 0.0071 | 9 | miR-486 | 1.31 | 0.0093 |
| 3 | miR-133a | 1.6 | 0.0002 | 10 | miR-1952 | 1.28 | 0.0130 |
| 4 | miR-1224 | 1.46 | 0.0138 | 11 | miR-22 | 1.25 | 0.0094 |
| 5 | miR-378 | 1.43 | 0.0063 | 12 | miR-30c | 1.19 | 0.0208 |
| 6 | miR-29c | 1.39 | 0.0032 | 13 | miR-322 | 1.15 | 0.0062 |
| 7 | miR-720 | 1.35 | 0.0043 | 14 | miR-151-5p | 1.11 | 0.0486 |
| **Downregulated miRNAs** | | | | | | | |
| S. No | miRNAs | Fold change | p-value | S. No | miRNAs | Fold change | p-value |
| 1 | miR-205 | -1.47 | 0.0007 | 24 | miR-431 | -1.3 | 0.0417 |
| 2 | miR-323-3p | -1.47 | 0.0039 | 25 | miR-539 | -1.29 | 0.0388 |
| 3 | miR-804 | -1.39 | 0.0088 | 26 | miR-669a | -1.29 | 0.0425 |
| 4 | miR-505 | -1.39 | 0.0138 | 27 | miR-2137 | -1.29 | 0.0428 |
| 5 | miR-202-3p | -1.38 | 0.0276 | 28 | miR-33 | -1.27 | 0.0174 |
| 6 | miR-433 | -1.38 | 0.0291 | 29 | miR-210 | -1.27 | 0.022 |
| 7 | miR-2145 | -1.37 | 0.0385 | 30 | miR-146a | -1.26 | 0.0102 |
| 8 | miR-1941-5p | -1.37 | 0.0423 | 31 | miR-20a+miR-20b | -1.26 | 0.0146 |
| 9 | miR-468 | -1.37 | 0.0458 | 32 | miR-154 | -1.25 | 0.0173 |
| 10 | miR-380-5p | -1.36 | 0.0313 | 33 | miR-2141 | -1.25 | 0.0434 |
| 11 | miR-654-3p | -1.36 | 0.0483 | 34 | miR-24 | -1.24 | 0.0005 |
| 12 | miR-1902 | -1.35 | 0.0327 | 35 | miR-466g | -1.24 | 0.0054 |
| 13 | miR-2138 | -1.34 | 0.0341 | 36 | miR-125a-3p | -1.24 | 0.0358 |
| 14 | miR-290-5p | -1.33 | 0.0154 | 37 | miR-423-5p | -1.23 | 0.0318 |
| 15 | miR-434-5p | -1.33 | 0.0223 | 38 | miR-484 | -1.23 | 0.032 |
| 16 | miR-M1-2 | -1.33 | 0.045 | 39 | miR-26a | -1.22 | 0.0014 |
| 17 | miR-214 | -1.32 | 0.0069 | 40 | miR-376b | -1.2 | 0.0279 |
| 18 | miR-714 | -1.32 | 0.0411 | 41 | miR-96 | -1.2 | 0.0377 |
| 19 | miR-695 | -1.31 | 0.0252 | 42 | miR-434-3p | -1.18 | 0.0481 |
| 20 | miR-674 | -1.31 | 0.0289 | 43 | miR-101b | -1.17 | 0.0429 |
| 21 | miR-683 | -1.31 | 0.0436 | 44 | miR-532-5p | -1.16 | 0.0061 |
| 22 | miR-31 | -1.3 | 0.0139 | 45 | miR-99b | -1.16 | 0.0116 |
| 23 | miR-421 | -1.3 | 0.0391 | 46 | miR-106a+miR-17 | -1.15 | 0.0371 |

**TABLE S4** *F. nucleatum* infection-induced upregulated and downregulated miRNAs in 8 weeks infected female vs male comparison.

| **Upregulated miRNAs** | | | | | | | |
| --- | --- | --- | --- | --- | --- | --- | --- |
| **S. No** | **miRNA** | **Fold change** | **p-value** | **S. No** | **miRNA** | **Fold change** | **p-value** |
| 1 | miR-206 | 2 | 0.0113 | 7 | miR-290-3p | 1.42 | 0.0394 |
| 2 | miR-1224 | 1.61 | 0.0217 | 8 | miR-7a | 1.39 | 0.0111 |
| 3 | miR-770-5p | 1.58 | 0.0443 | 9 | miR-331-3p | 1.38 | 0.0334 |
| 4 | miR-2138 | 1.53 | 0.0345 | 10 | miR-199a-3p | 1.24 | 0.0235 |
| 5 | miR-205 | 1.42 | 0.0174 | 11 | mmu-let-7b | 1.21 | 0.0065 |
| 6 | miR-210 | 1.42 | 0.0242 | 12 | miR-1944 | 1.17 | 0.0292 |
| **Downregulated miRNAs** | | | | | | | |
| S. No | miRNA | Fold change | p-value | S. No | miRNA | Fold change | p-value |
| 1 | miR-376a | -1.64 | 0.0117 | 7 | miR-145 | -1.3 | 0.0107 |
| 2 | miR-2183 | -1.47 | 0.0047 | 8 | mmu-let-7f | -1.29 | 0.0493 |
| 3 | miR-410 | -1.43 | 0.0017 | 9 | miR-30b | -1.28 | 0.0128 |
| 4 | miR-450a-5p | -1.37 | 0.033 | 10 | miR-126-5p | -1.25 | 0.0358 |
| 5 | miR-127 | -1.36 | 0.035 | 11 | miR-143 | -1.25 | 0.0362 |
| 6 | miR-328 | -1.34 | 0.0061 | 12 | miR-350 | -1.21 | 0.0286 |

**TABLE S5** *F. nucleatum* infection-induced upregulated and downregulated miRNAs in 16 weeks infected female vs male comparison.

| **Upregulated miRNAs** | | |
| --- | --- | --- |
| miRNA | Fold change | p-value |
| miR-152 | 1.36 | 0.0432 |
| miR-125b-5p | 1.3 | 0.0484 |
| miR-495 | 1.25 | 0.0327 |
| miR-28 | 1.15 | 0.0403 |
| miR-675-3p | 1.13 | 0.0435 |
| **Downregulated miRNAs** | | |
| miRNA | Fold change | p-value |
| miR-375 | -2.47 | 0.0458 |
| miR-223 | -2.16 | 0.0432 |
| miR-320 | -1.54 | 0.0205 |
| miR-142-3p | -1.52 | 0.0489 |
| miR-142-5p | -1.45 | 0.03 |
| miR-669g | -1.41 | 0.0292 |
| miR-20a+miR-20b | -1.29 | 0.0279 |
| miR-376a | -1.28 | 0.0313 |
| miR-362-3p | -1.27 | 0.011 |
| miR-23a | -1.25 | 0.0048 |
| miR-204 | -1.22 | 0.0229 |
| miR-106a+miR-17 | -1.19 | 0.0049 |
| miR-210 | -1.19 | 0.0337 |
| miR-425 | -1.16 | 0.0219 |

**TABLE S6** miRNAs and targeting genes in the bacterial invasion of epithelial cells pathway.

| **Gene name** | **Gene Ensemble ID** | **miRNA** |
| --- | --- | --- |
| *Pxn* | 00000029528 | miR-345-5p |
| *Cblb* | 00000022637 | miR-361-5p |
| *Rhoa* | 00000007815 |  |
| *Rac1* | 00000001847 |  |
| *Arpc1b* | 00000029622 | miR-218-5p |
| *Elmo1* | 00000041112 |  |
| *Shc4* | 00000035109 |  |
| *Pik3r1* | 00000041417 |  |

**TABLE S7** List of upregulated miRNAs (8 weeks *F. nucleatum* infection) and their associated genes with Gene Ensembl IDs in the Pathways of Cancer.

| **S. No** | **miRNA** | **Fold Change** | **Genes** | **Gene Ensembl ID** | **Reference**  **PMID** | **S. No** | **miRNA** | **Fold Change** | **Genes** | **Gene Ensembl ID** | **Reference**  **PMID** |
| --- | --- | --- | --- | --- | --- | --- | --- | --- | --- | --- | --- |
| 1 | miR-126-5p | 1.12 | *Axin1* | 00000024182 | 36356835 | 28 | miR-24-3p | 1.17 | *Fasl* | 00000000817 | 15962391 |
| 2 |  |  | *Fgf7* | 00000027208 | 31183192 | 29 |  |  | *Wnt4* | 00000036856 | 33222684 |
| 3 |  |  | *Wnt16* | 00000029671 | -- | 30 |  |  | *Tcf7* | 00000000782 | 32403323 |
| 4 |  |  | *Runx1t1* | 00000006586 | 30499136 | 31 |  |  | *Rala* | 00000008859 | 36466919 |
| 5 |  |  | *Sos1* | 00000024241 | 37042566 | 32 |  |  | *Egfr* | 00000020122 | 26468449 |
| 6 |  |  | *Traf3* | 00000021277 | 34257589 | 33 |  |  | *Adcy1* | 00000020431 | 33744851 |
| 7 |  |  | *Shh* | 00000002633 | 33498528 | 34 |  |  | *Fgf15* | 00000031073 | 28969019 |
| 8 | miR-99b-5p | 1.14 | *Nkx3-1* | 00000022061 | -- | 35 |  |  | *Mecom* | 00000027684 | 36609474 |
| 9 |  |  | *Mtor* | 00000028991 | 35883111 | 36 |  |  | *Wnt8b* | 00000036961 | 32923386 |
| 10 | miR-26a-5p | 1.23 | *Pik3r3* | 00000028698 | 32973127 | 37 |  |  | *Ret* | 00000030110 | 33906292 |
| 11 |  |  | *Ep300* | 00000055024 | 12385008 | 38 |  |  | *Fzd5* | 00000045005 | 37595345 |
| 12 |  |  | *Gsk3b* | 00000022812 | 29568361 | 39 | miR-361-5p | 1.3 | *Cblb* | 00000022637 | 30029779 |
| 13 |  |  | *Cblb* | 00000022637 | 30029779 | 40 |  |  | *Cul2* | 00000024231 | 35027503 |
| 14 |  |  | *Skp2* | 00000054115 | 35301297 | 41 |  |  | *Fgf7* | 00000027208 | 31183192 |
| 15 |  |  | *Ptgs2* | 00000032487 | 26957558 | 42 |  |  | *Rhoa* | 00000007815 | 29206819 |
| 16 |  |  | *Cks2* | 00000062248 | 24137409 | 43 |  |  | *Vegfa* | 00000023951 | 35328794 |
| 17 |  |  | *Hgf* | 00000028864 | 28423584 | 44 |  |  | *Arhgef12* | 00000059495 | 37228491 |
| 18 |  |  | *Ralgds* | 00000026821 | 29196555 | 45 |  |  | *Rac1* | 00000001847 | 32178475 |
| 19 |  |  | *Zbtb16* | 00000066687 | 24348178 | 46 |  |  | *Wnt7a* | 00000030093 | 33791195 |
| 20 |  |  | *Ret* | 00000030110 | 33906292 | 47 |  |  | *Col4a6* | 00000031273 | 32945508 |
| 21 |  |  | *Il6* | 00000025746 | 23136553 | 48 |  |  | *Hsp90aa1* | 00000021270 | 34827586 |
| 22 |  |  | *Plcb1* | 00000051177 | 7893368 | 49 |  |  | *Crebbp* | 00000022521 | 33708426 |
| 23 |  |  | *Pten* | 00000013663 | 24475377 | 50 | miR-193a-3p | 1.21 | *Sos2* | 00000034801 | -- |
| 24 |  |  | *Bcr* | 00000009681 | 29438985 | 41 |  |  | *Fgf1* | 00000036585 | 21356388 |
| 25 |  |  | *Gna13* | 00000020611 | 25193986 | 52 |  |  | *Lef1* | 00000027985 | 34474344 |
| 26 |  |  | *Ccdc6* | 00000048701 | 26078337 | 53 |  |  | *Runx1t1* | 00000006586 | 30499136 |
| 27 |  |  | *Crebbp* | 00000022521 | 33708426 | 54 |  |  | *Arhgef12* | 00000059495 | 37228491 |
| Nil |  |  |  |  |  | 55 |  |  | *Kras* | 00000030265 | 25713627 |
| Nil |  |  |  |  |  | 56 |  |  | *Pten* | 00000013663 | 24475377 |
| Nil |  |  |  |  |  | 57 |  |  | *Kit* | 00000005672 | 35842424 |

**TABLE S8** List of upregulated miRNAs (16 weeks *F. nucleatum* infection) and their associated genes with Gene Ensembl IDs in the Pathways of Cancer.

| **miRNA** | **Fold Change** | **Gene name** | **Gene Ensemble ID** | **Reference (PMID)** |
| --- | --- | --- | --- | --- |
| miR-99b-5p | 1.15 | *Nkx3-1* | 00000022061 | -- |
|  |  | *Mtor* | 00000028991 | 35883111 |
| miR-218-5p | 1.15 | *Pik3r1* | 00000041417 | 21723986 |
|  |  | *Fgf12* | 00000022523 | -- |
|  |  | *Adcy1* | 00000020431 | 33744851 |
|  |  | *Plcg1* | 00000016933 | 29464031 |
|  |  | *Mecom* | 00000027684 | 36609474 |
|  |  | *Mitf* | 00000035158 | -- |
|  |  | *Gng3* | 00000071658 | -- |
|  |  | *Hsp90ab1* | 00000023944 | https://doi.org/10.1515/oncologie-2023-0602 |
|  |  | *Wnt2b* | 00000027840 | 31121307 |
|  |  | *Arhgef12* | 00000059495 | 37228491 |
| miR-361-5p | 1.19 | *Cblb* | 00000022637 | 30029779 |
|  |  | *Cul2* | 00000024231 | 35027503 |
|  |  | *Fgf7* | 00000027208 | 31183192 |
|  |  | *Rhoa* | 00000007815 | 29206819 |
|  |  | *Vegfa* | 00000023951 | 35328794 |
|  |  | *Arhgef12* | 00000059495 | 37228491 |
|  |  | *Rac1* | 00000001847 | 32178475 |
|  |  | *Wnt7a* | 00000030093 | 33791195 |
|  |  | *Col4a6* | 00000031273 | 32945508 |
|  |  | *Hsp90aa1* | 00000021270 | 34827586 |
|  |  | *Crebbp* | 00000022521 | 33708426 |
| mmu-let-7a-5p &  mmu-let-7f-5p | 1.28  1.16 | *Bcl2l1* | 00000007659 | 22009326 |
|  |  | *Wnt16* | 00000029671 | -- |
|  |  | *Wnt9b* | 00000018486 | 29393416 |
|  |  | *Ccne2* | 00000028212 | 38515149 |
|  |  | *Fgf11* | 00000042826 | 37805185 |
|  |  | *Casp3* | 00000031628 | 29524226 |
|  |  | *Tgfbr1* | 00000007613 | 19147584 |
|  |  | *Runx1t1* | 00000006586 | 30499136 |
|  |  | *Ptch2* | 00000028681 | 34573430 |
|  |  | *Nras* | 00000027852 | 38800532 |
|  |  | *Wnt9a* | 00000000126 | 27049382 |
|  |  | *Araf* | 00000001127 | 37020037 |
|  |  | *Il6* | 00000025746 | 23136553 |
|  |  | *Col4a2* | 00000031503 | -- |
| mmu-let-7f-5p | 1.16 | *Mapk8* | 00000021936 | 23027623 |

**TABLE S9** miRTarBase analysis of upregulated DE microRNAs and their target genes in 8 weeks of *F. nucleatum* infection.

| **miRTarBase ID** | **Name of miRNA** | **Target gene** |
| --- | --- | --- |
| MIRT584077 | mmu-miR-361-5p | *Ctbp2* |
| MIRT583933 | mmu-miR-361-5p | *Dnmt3a* |
| MIRT597100 | mmu-miR-361-5p | *Tagln2* |
| MIRT595620 | mmu-miR-361-5p | *Tfam* |
| MIRT600573 | mmu-miR-361-5p | *Nr5a2* |
| MIRT594155 | mmu-miR-361-5p | *Vps26a* |
| MIRT603832 | mmu-miR-361-5p | *Kcnk6* |
| MIRT595884 | mmu-miR-361-5p | *Gid4* |
| MIRT595624 | mmu-miR-361-5p | *Nol7* |
| MIRT594201 | mmu-miR-361-5p | *Zfp120* |
| MIRT594781 | mmu-miR-361-5p | *Ssr1* |
| MIRT583847 | mmu-miR-361-5p | *Eea1* |
| MIRT595967 | mmu-miR-361-5p | *Tnpo1* |
| MIRT577458 | mmu-miR-361-5p | *Tspan18* |
| MIRT604433 | mmu-miR-361-5p | *Endov* |
| **List of other miRNAs and their total miRTarBase ID, Target gene details** | | |
| miRNA | # of MiRTarBase IDs | # of Target genes |
| miR-26a-5p | 426 | 426 |
| miR-193a-3p | 8 | 8 |
| miR-126-5p | 55 | 55 |
| miR-324-5p | 12 | 12 |
| miR-24-3p | 375 | 375 |
| miR-99b-5p | 4 | 4 |

We used mmu-miR-361-5p as an example for an upregulated DE miRNA during 8 weeks of infection in identifying the target genes using the miRTarBase. Each miRNA has different target genes each with a specific MiRTarBase ID. *F. nucleatum*-infection induced DE upregulated mmu-miR-361-5p has 15 different target genes with 15 different MiRTarBase IDs as stated in the table. The other 5 upregulated miRNAs and their # of target genes are stated in a separate table.

**TABLE S10** miRTarBase analysis of upregulated DE microRNAs and their target genes in 16 weeks of *F. nucleatum* infection.

| **MiRTarBase ID** | **miRNA** | **Target gene** |
| --- | --- | --- |
| MIRT004802 | mmu-let-7a-5p | *Lin28a* |
| MIRT438710 | mmu-let-7a-5p | *IL6* |
| MIRT001016 | mmu-let-7a-5p | *Hoxa9* |
| MIRT004728 | mmu-let-7a-5p | *Trim71* |
| MIRT601176 | mmu-let-7a-5p | *Atp2b2* |
| MIRT605687 | mmu-let-7a-5p | *Ifnar1* |
| MIRT594646 | mmu-let-7a-5p | *Meis2* |
| MIRT578155 | mmu-let-7a-5p | *Nf2* |
| MIRT579375 | mmu-let-7a-5p | *Apc2* |
| MIRT579029 | mmu-let-7a-5p | *Cts8* |
| MIRT586732 | mmu-let-7a-5p | *Lins* |
| MIRT596613 | mmu-let-7a-5p | *Zfp444* |
| MIRT582399 | mmu-let-7a-5p | *Msi2* |
| MIRT583580 | mmu-let-7a-5p | *Fbxl14* |
| MIRT592941 | mmu-let-7a-5p | *Armc2* |
| MIRT597968 | mmu-let-7a-5p | *Nup214* |
| MIRT578709 | mmu-let-7a-5p | *Gnl3l* |
| MIRT601461 | mmu-let-7a-5p | *Tnfrsf26* |
| MIRT592593 | mmu-let-7a-5p | *Mup11* |
| MIRT592561 | mmu-let-7a-5p | *Mup17* |
| MIRT053615 | mmu-let-7a-5p | *ACVR1B* |
| MIRT737100 | mmu-let-7a-5p | *Prdm1* |
| MIRT002019 | mmu-let-7a-5p | *Hmga2* |
| MIRT004675 | mmu-let-7a-5p | *Il13* |
| MIRT733431 | mmu-let-7a-5p | *Igf1* |
| MIRT733432 | mmu-let-7a-5p | *Il1a* |
| MIRT732594 | mmu-let-7a-5p | *Stat3* |
| MIRT733433 | mmu-let-7a-5p | *Tgfb1* |
| **List of remaining 5 upregulated miRNAs and their total miRTarBase ID, Target gene details** | | |
| miRNA | # of MiRTarBase IDs | # of Target genes |
| miR-127-3p | 10 | 10 |
| miR-361-5p | 15 | 15 |
| miR-345-5p | 10 | 10 |
| mmu-let-7f-5p | 16 | 16 |
| mmu-99b-5p | 4 | 4 |
| miR-218-5p | 20 | 20 |

The mmu-let-7a-5p an upregulated DE miRNA during 16 weeks of *F. nucleatum*-infection has 28 different target genes with 28 different MiRTarBase IDs as stated in the table. The other 6 upregulated miRNAs and their # of target genes are stated in a separate table.

**APPENDICES**

**Appendix S1: Hyperparameters for machine learning models**

Information about the hyperparameters used for the machine models are available at:

- XGBoost (XGB):
  https://xgboost.readthedocs.io/en/stable/parameter.html
- Random Forest Classifier (RFC):
  https://scikit-learn.org/stable/modules/generated/sklearn.ensemble.RandomForestClassifier.html
- Logistic Regression (LR):
  https://scikit-learn.org/stable/modules/generated/sklearn.linear_model.LogisticRegression.html
- C-Support Vector Classifier:
  https://scikit-learn.org/stable/modules/generated/sklearn.svm.SVC.html#sklearn.svm.SVC
- Multilayer Perceptron (MLP):
  https://scikit-learn.org/stable/modules/generated/sklearn.neural_network.MLPClassifier.html

The following tables provide information about the hyperparameter settings used during the execution of each machine learning model on the data from 8-week, 16-week, and 8- and 16-week combined cohorts of mice.

**eXtreme Gradient Boost (XGBoost)**

| **Parameter** | **8-week data** | **16-week data** | **8- and 16-week data** |
| --- | --- | --- | --- |
| booster | gbtree | gbtree | gbtree |
| colsample_bytree | 0.6 | 0.4 | 0.6 |
| gamma | 0.2 | 0.3 | 0.2 |
| learning_rate | 0.5 | 0.1 | 0.5 |
| max_depth | 40 | 200 | 40 |
| n_estimators | 475 | 275 | 475 |
| objective | binary:logistic | binary:logistic | binary:logistic |
| random_state | 0 | 0 | 0 |

**Random Forest Classifier**

| **Parameter** | **8-week data** | **16-week data** | **8- and 16-week data** |
| --- | --- | --- | --- |
| bootstrap | False | False | True |
| criterion | gini | entropy | gini |
| max_depth | 90 | 20 | 20 |
| max_features | None | None | None |
| min_samples_leaf | 2 | 4 | 4 |
| min_samples_split | 8 | 2 | 8 |
| n_estimators | 290 | 20 | 160 |
| oob_score | False | False | False |
| random_state | 0 | 0 | 0 |

**Logistic regression**

| **Parameter** | **8-week data** | **16-week data** | **8- and 16-week data** |
| --- | --- | --- | --- |
| C | 0.1 | 10 | 10 |
| max_iter | 50 | 50 | 100 |
| penalty | L2 | L2 | L2 |
| solver | newton-cg | newton-cg | lbfgs |

**C-Support Vector Classifier**

| **Parameter** | **8-week data** | **16-week data** | **8- and 16-week data** |
| --- | --- | --- | --- |
| C | 0.1 | 0.1 | 0.1 |
| gamma | 1 | 1 | 1 |
| kernel | poly | poly | poly |

**Multilayer Perceptron**

| **Parameter** | **8-week data** | **16-week data** | **8- and 16-week data** |
| --- | --- | --- | --- |
| activation | relu | relu | relu |
| alpha | 0.01 | 0.01 | 0.01 |
| hidden_layer_sizes | 256, 128 | 400, 200 | 400, 200 |
| learning_rate | adaptive | constant | constant |
| solver | adam | adam | adam |
